# Covalent Organic Framework Composites: Synthesis and Analytical Applications

**DOI:** 10.3390/molecules25225404

**Published:** 2020-11-18

**Authors:** Jenni J. Jarju, Ana M. Lavender, Begoña Espiña, Vanesa Romero, Laura M. Salonen

**Affiliations:** 1 International Iberian Nanotechnology Laboratory (INL), Av. Mestre José Veiga, 4715-330 Braga, Portugal; jenni.jarju@inl.int (J.J.J.); micaela.brandaolavender@wur.nl (A.M.L.); begona.espina@inl.int (B.E.); 2 Department of Food and Analytical Chemistry, Marine Research Center (CIM), University of Vigo, As Lagoas, Marcosende, 36310 Vigo, Spain

**Keywords:** covalent organic frameworks, composite, analytical applications, separation, sensing

## Abstract

In the recent years, composite materials containing covalent organic frameworks (COFs) have raised increasing interest for analytical applications. To date, various synthesis techniques have emerged that allow for the preparation of crystalline and porous COF composites with various materials. Herein, we summarize the most common methods used to gain access to crystalline COF composites with magnetic nanoparticles, other oxide materials, graphene and graphene oxide, and metal nanoparticles. Additionally, some examples of stainless steel, polymer, and metal-organic framework composites are presented. Thereafter, we discuss the use of these composites for chromatographic separation, environmental remediation, and sensing.

## 1. Introduction

Covalent organic frameworks (COFs) are crystalline, porous materials formed by the self-assembly of organic building blocks [1,2,3]. They have attracted a lot of interest due to their unique properties, such as large surface area, ordered channels, high porosity, tunable structure through pre-selection of the building blocks, easy functionalization, and good thermal and chemical stability. To tackle challenges in sample treatment, separation, and sensing processes, COF materials have attracted much attention in the last few years, offering highly efficient tools for analytical applications.

In order to impart additional functionality to these materials, COFs can be combined with other functional materials, such as metal and SiO_2_ nanoparticles or graphene, or grown on substrates, such as alumina or stainless steel. Such COF composite materials open various further possibilities of exploitation for a wide variety of applications. In the last few years, development of synthetic approaches to obtain bulk COFs and COF-based composites with favorable characteristics, such as large surface area, high crystallinity, high chemical and thermal stability, durability, etc., has received increasing attention. This fact, together with the rapid development of analytical applications of COF materials, has resulted in different reviews since 2017 focusing on COF synthesis [3,4], design [5], and applications as sensing platforms [6,7,8,9,10], adsorbents in sample pretreatment [11,12,13,14,15,16,17], stationary phases in chromatographic separation [18,19,20], and novel tools in water treatment [21,22]. However, since research on COF composites in the analytical chemistry field is relatively recent as compared to bulk COFs, to the best of our knowledge, no reviews have focused on COF composites developed for analytical applications. To address this gap, herein we aim at providing an overview of different synthesis approaches that have been shown to successfully result in crystalline and porous COF composites. Then, we review their applications in different areas of analytical chemistry by covering examples of sensing, environmental remediation, extraction, and separation processes, and highlighting the role of each component in the composites.

The synthesis Section 2 is grouped by material type, starting with iron oxide composites, by far the most reported COF composite, and moving to other oxide materials, and carbon-based, metallic, and finally polymeric and metal-organic framework (MOF) composites. Section 3 on the application of these composites is divided into separation and sensing. The use of COF composites in solid sorbent-based extraction procedures for sample pretreatment and for environmental remediation has driven especially important advances, overcoming in some cases the pitfalls of conventional sorbents, such as low selectivity and low capacity for complex samples [11]. Finally, we will conclude in Section 4 by considering the future perspectives and challenges of COF composite materials. As an aid to the readers, abbreviations for the most commonly appearing building blocks in this review are presented in Table 1.

## 2. Synthesis of COF Composites

### 2.1. Iron Oxide—COF Composites

Magnetic composites are the most reported COF composite materials. Magnetic nanoparticles (NPs) are attractive for a wide range of applications due to their remarkable magnetic properties, stability, biocompatibility, and fast separation using an external magnetic field [23]. The majority of magnetic COF composites contain iron oxide, Fe_3_O_4_, as the magnetic NP, although a few other examples with NiFe_2_O_4_ [24] and CuFe_2_O_4_ [25] NPs have been reported. Superparamagnetic iron oxide NPs are Fe_3_O_4_ particles with cores below 100 nm in diameter [26]. These particles become magnetized up to their saturation magnetization upon application of an external magnetic field, and on removal of the magnetic field, they no longer exhibit magnetic interactions. Therefore, gaining access to nanocomposites that combine the functionality of COFs with magnetic separability is of great interest.

In a typical procedure, magnetite is prepared first, followed by surface functionalization with anchoring groups, such as amino functionalities. Thereafter, one of the COF building blocks can be attached to the anchoring groups prior to COF growth, a step that can be crucial to obtaining a crystalline COF composite. Finally, the COF is typically grown under solvothermal conditions.

**Figure 1:**
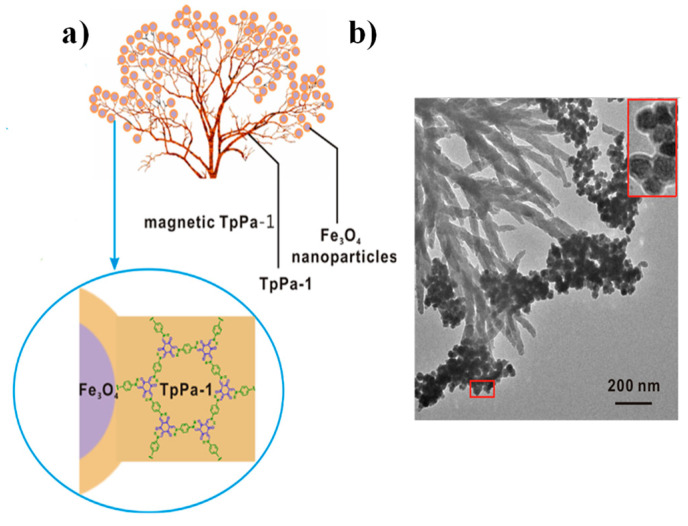
(**a**) The structure of the bouquet-like magnetic TpPa-1 sorbent, and (**b**) TEM image of magnetic TpPa-1. Reprinted with permission from [27]. Copyright (2017) American Chemical Society.

A bouquet-shaped Fe_3_O_4_-TpPa-1 nanocomposite [27] was developed by a synthetic procedure starting with amino-functionalized Fe_3_O_4_ NPs (NH_2_–Fe_3_O_4_ NPs) with a size of ≈25 nm (Figure 1a). NH_2_–Fe_3_O_4_ NPs were prepared using FeCl_3_·6H_2_O, sodium acetate, and 1,6-hexamethylenediamine by first stirring at 50 °C and then heating in an autoclave at 198 °C for 6 h [28], after which their surface was modified with 1,3,5-triformylphloroglucinol (Tp) [27]. The COF composite was prepared by room-temperature solution-phase method from Tp and *p*-phenylenediamine (Pa-1) in ethanol, and the resulting composite featured crystallinity, a specific surface area of 248 m^2^·g^−1^ (lower than that observed for bulk COF), and superparamagnetic nature. The transmission electron microscopy (TEM) image (Figure 1b) showed that interconnected nanofibers of TpPa-1 sprouted from clustered NH_2_-Fe_3_O_4_ nanoparticles. In another study, Espiña, Salonen, and co-workers [29] developed a synthesis protocol to prepare magnetic COF composites by installing the anchoring groups on the magnetic nanoparticles already during the NP synthesis. The mTpBD-Me_2_ composites were synthesized via a facile three-step procedure, starting with dopamine (DOPA) being introduced as capping ligand during co-precipitation to produce spherical Fe_3_O_4_@DOPA NPs with free amino groups and an average diameter of 5 nm. Fe_3_O_4_@DOPA NPs were then pre-functionalized with Tp to provide the NP surfaces with nucleation points for directed growth of COF around the nanoparticles. TpBD-Me_2_ was grown onto the NPs under solvothermal conditions using Tp and *o*-tolidine as building blocks. The crystalline composite exhibited, with 538 m^2^·g^−1^, no loss in Brunauer-Emmett-Teller (BET) surface area as compared to the bulk COF material and was easily collected using an external magnetic field. In TEM images small Fe_3_O_4_ aggregates were observed encapsulated within a shell of COF. In a later study [30] the composite was prepared on 0.5 g scale, maintaining both crystallinity and porosity.

An alternative to the most common solvothermal synthesis was offered by Liao, Thomas, and co-workers [31], who combined mechanochemical and crystallization approaches to obtain COF-LZU1 composites. First, mechanical grinding of 1,3,5-triformylbenzene (TFB), Pa-1, and commercially available Fe_3_O_4_ followed by Soxhlet extraction led to Fe_3_O_4_/amorphous polymer network. The following mild crystallization reaction in the presence of a small amount of 1,4-dioxane/mesitylene/acetic acid at 70 °C for 5 days yielded a crystalline COF composite with a surface area of 872 m^2^·g^−1^, which is more than double that reported for the bulk COF-LZU1 material. The composites consisted of aggregated microparticles covered by a 15–35 nm thick COF shell with continuous and smooth appearance. This method was also applied to the synthesis of COF composites with Co_3_O_4_ and NiO.

Another example of a mechanochemical method for magnetic COF (MCOF) composite synthesis was presented by Shuai, Huang, and co-workers [32], where first, amino-functionalized silica-coated Fe_3_O_4_@SiO_2_ nanoparticles (MNP–NH_2_) were prepared following a solvothermal method [33]. For amino functionality incorporation [32], the Fe_3_O_4_ NPs were modified sequentially with tetraethyl orthosilicate (TEOS) and 3-aminopropyl trimethoxysilane (APTMS) to give MNP–NH_2_. For introduction of the COF, MNP-NH_2_ (50, 100, 150, and 200 mg for MCOF-1, MCOF-2, MCOF-3, and MCOF-4, respectively) were ground in a mortar for 5 min with *p*-toluenesulfonic acid monohydrate (PTSA), Tp, and Pa-1, and heated to 170 °C during 1 min. X-ray diffraction (XRD) confirmed the crystallinity of the MCOFs and the materials featured surface areas ranging from 122 to 352 m^2^·g^−1^. TEM imaging of MCOF-2 showed MNP–NH_2_ to be encapsulated within the COF.

In a recent study, a simple and rapid coprecipitation method was used to prepare COF-(TpBD)/Fe_3_O_4_ for solid-phase extraction [34]. First, TpBD was synthesized by solvothermal method, delaminated by mechanical grinding to nanosheets, and then added to a dispersion of FeCl_3_·H_2_O and FeSO_4_·7H_2_O in water. The suspension was stirred at 80 °C, followed by addition of 30% aqueous ammonia. The TEM images of COF-(TpBD)/Fe_3_O_4_ showed that Fe_3_O_4_ NPs were spherical in shape, had a size of <50 nm, and were embedded in TpBD nanosheets. In addition, powder XRD studies demonstrated that TpBD retained its crystallinity during the coprecipitation process.

**Figure 2:**
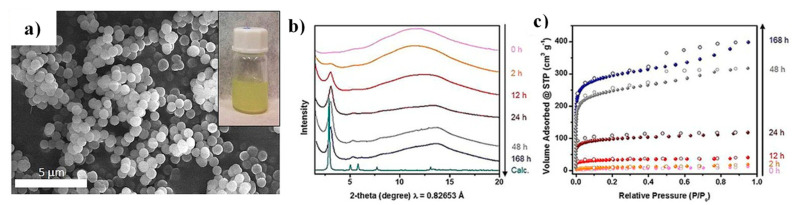
(**a**) Field emission scanning electron microscopy (FESEM) image of c-1 spheres, and evolution of the crystallinity (**b**) and porosity (**c**) of the composite during the amorphous-to-crystalline transformation. Reprinted with permission from [35]. Copyright (2017) Wiley-VCH Verlag GmbH and Co. KGaA, Weinheim.

A two-step strategy enabling the encapsulation of several types of NPs, including Fe_3_O_4_, into crystalline imine COF spheres, was reported by Maspoch, Zamora, and co-workers [35]. First, COF building blocks TFB and 1,3,5-tris(4-aminophenyl)benzene (TAPB) were separately dissolved in a suspension of Fe_3_O_4_ NPs (9.8 ± 3.9 nm) in acetone/acetic acid. To encapsulate the NPs in a polymer, the resulting solutions were combined and ultrasonicated for 1 h, and the precipitate was collected to yield amorphous imine-based Fe_3_O_4_@a-1 spheres. These spheres were then subjected to solvothermal COF growth conditions for 7 days. High-angle annular dark-field scanning transmission electron microscopy (HAADF-STEM) and powder XRD studies of the samples revealed the successful formation of crystalline spherical Fe_3_O_4_@c-1 NPs with Fe_3_O_4_ located mostly in the center, with BET surface area of 880 m^2^·g^−1^ (Figure 2).

**Figure 3:**
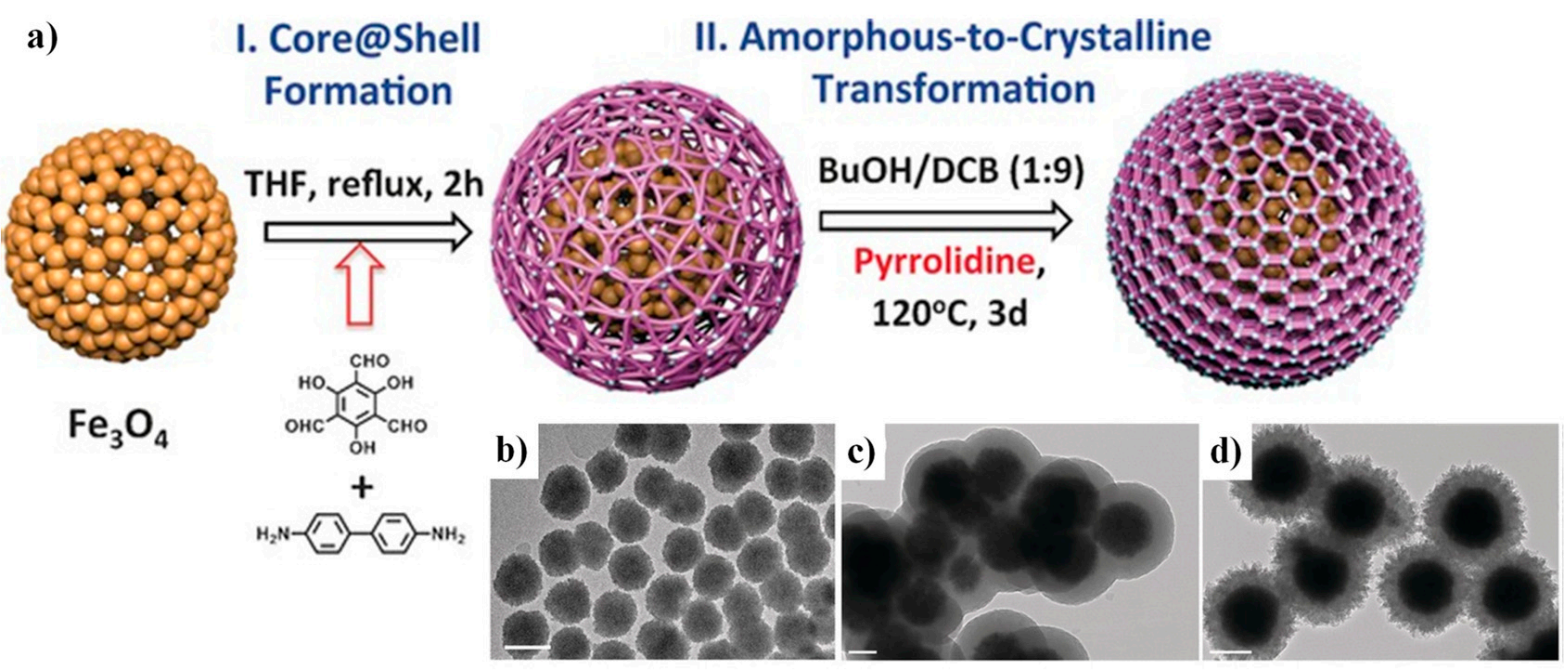
(**a**) The preparation of imine-linked COF composite microspheres via the amorphous-to-crystalline conversion process. High-resolution TEM images of (**b**) Fe_3_O_4_ nanoparticles, (**c**) amorphous Fe_3_O_4_@polyimine, and (**d**) Fe_3_O_4_@COF(TpBD) microspheres. Reprinted with permission from [36]. Copyright (2016) Wiley-VCH Verlag GmbH and Co. KGaA, Weinheim, Germany.

In a similar amorphous-to-crystalline approach, core-shell microspheres were prepared by template-mediated precipitation polymerization of an Fe_3_O_4_ nanocluster core with an amorphous polyimine shell, followed by crystallization to COF under solvothermal conditions (Figure 3) [36]. The employed citrate-stabilized Fe_3_O_4_ nanoclusters [37] had ample carboxylate groups on the surface to offer high dispersibility. In addition, these moieties also serve as anchoring groups to initiate the polyimine network formation through H-bonding interactions with the benzidine (BD) monomer. Thereafter, the amorphous polyimine network shell was formed via the template-controlled precipitation polymerization of BD and Tp. Subsequent treatment of the NPs under solvothermal conditions using pyrrolidine as catalyst resulted in highly crystalline Fe_3_O_4_@COF(TpBD) microspheres with a well-defined core-shell structure, narrow particle-size distribution, and uniform spherical shape with a high BET surface area of 1346 m^2^·g^−1^.

Core-shell nanostructures can have the advantages of hindering the aggregation of the magnetic cores and reducing losses of magnetism that stem from high organic content, while allowing surface modification. Using a layer-by-layer (LBL) assembly protocol, a COF shell was assembled on the surface of 1,6-hexamethylenediamine-functionalized Fe_3_O_4_-NH_2_ NPs [38] of 100 nm. First, 2,5-dimethoxyterephthalaldehyde (DMTP) was added to a suspension of Fe_3_O_4_-NH_2_ NPs in 1,4-dioxane with aqueous acetic acid and heated at 70 °C for 2 h. After washing, TAPB was added with aqueous acetic acid and the reaction mixture was again heated at 70 °C for 2 h followed by washing and drying to form Fe_3_O_4_–NH_2_@TAPB–DMTP–COFs. The resulting TAPB–DMTP–COF layer had a thickness of about 12 nm, the spherical NP structure was preserved, and no interconnectivity between the particles was observed. The composite showed high crystallinity and excellent performance as electrochemical sensor material with good sensitivity and recovery, high stability, low detection limit, and wide linear range due to the catalytic activity of Fe_3_O_4_ NPs and the high electroactive surface area of TAPB-DMTP-COF layer.

**Figure 4:**
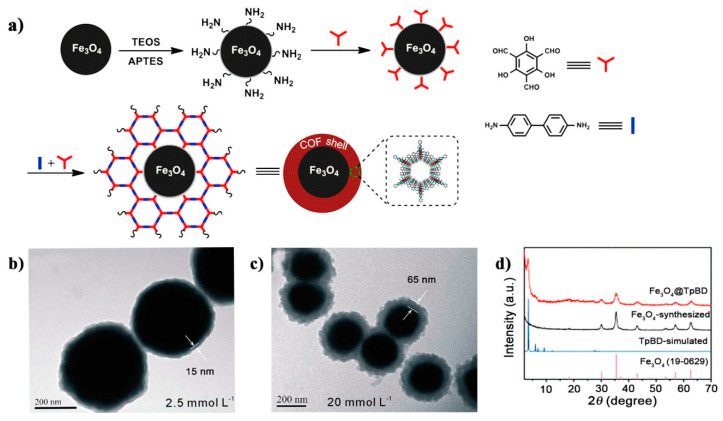
(**a**) Illustration of the monomer-mediated in situ growth of core-shell Fe_3_O_4_@TpBD. (**b,c**) TEM images of the monomer concentration effect on Fe_3_O_4_@TpBD. (**d**) The XRD patterns of Fe_3_O_4_@TpBD, Fe_3_O_4_, and simulated TpBD. Reproduced from [23] with permission from The Royal Society of Chemistry.

Controllable preparation of core-shell Fe_3_O_4_@TpBD nanospheres [23] was achieved by growing the COF on magnetic NPs functionalized by TEOS and (3-aminopropyl)triethoxysilane (APTES) (Figure 4a). The obtained Fe_3_O_4_-NH_2_ was pre-functionalized with Tp for subsequent in situ growth of a TpBD shell. Pre-functionalization of the nanosphere surface with Tp was crucial to obtain uniform crystalline COF shells, whereas the COF shell crystallinity and thickness were controlled by the solvents, concentration of building blocks, and reaction time. The thickness of the COF shell around the nanosphere was tuned from 15 to 65 nm by varying the concentration of COF monomers from 2.5 to 20 mmol·L^−1^ (Figure 4b,c).

### 2.2. Silicon Dioxide–COF Composites

COFs have been successfully used as stationary phases in gas chromatography and capillary electro-chromatography [39]. In high-performance liquid chromatography (HPLC), irregular shape, sub-micrometer size, or broad size distribution of pristine COFs may cause low column efficiency and column backpressure of the COF-packed columns. Growing a uniform COF shell on SiO_2_ microspheres is a promising approach to resolve the above-mentioned problems. There are three methods to prepare COF-SiO_2_ composites: (i) in situ growth strategy, (ii) layer-by-layer (LBL) method, and (iii) bottom-up strategy.

In situ growth strategy was employed to gain access to uniform core-shell COF@SiO_2_ microspheres with controllable thickness [39]. For the fabrication, spherical 5 µm SiO_2_-NH_2_ served as both the core and the amino group source for reaction with Tp to induce controllable TpBD shell growth. The resulting SiO_2_-Tp was further reacted with BD to form the TpBD shell on the silica core. The thickness of the TpBD shell can be easily controlled by adjusting the monomer concentrations. Only a few TpBD crystals were formed on TpBD@SiO_2_ when 0.10 mmol of Tp and 0.15 mmol of BD were used. Raising the amount of Tp to 0.30 mmol and the amount of BD to 0.45 mmol increased the shell thickness from 50 to 150 nm. Further increases in Tp and BD monomer amounts, to 0.50 and 0.75 mmol, respectively, did not lead to clear improvement in the shell thickness, indicating the full occupation of the amino groups on SiO_2_-NH_2_ by Tp. In the powder XRD, the peak intensities of TpBD@SiO_2_-0.10, TpBD@SiO_2_-0.30, and TpBD@SiO_2_-0.50 increased, revealing the controllable synthesis of TpBD@SiO_2_ microspheres.

**Figure 5:**
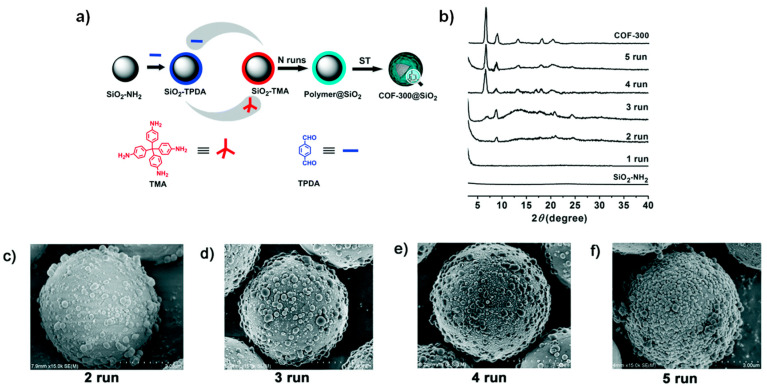
(**a**) Schematic illustration of the synthesis of COF-300@SiO_2_ via LBL method; (**b**) powder XRD patterns of COF-300@SiO_2_ after different reaction runs; (**c**–**f**) SEM images of COF-300@SiO_2_ after (**c**) 2, (**d**) 3, (**e**) 4, and (**f**) 5 runs. Reproduced from [40] with permission from The Royal Society of Chemistry.

The LBL method was first reported for the preparation of a 3D COF on SiO_2_ [40]. Aminosilica (SiO_2_-NH_2_) nanoparticles with 5 µm size were consecutively refluxed with terephthalaldehyde (TPDA) and tetra(4-anilyl)methane (TAM) for several cycles to obtain polymer@SiO_2_, which was converted into ordered COF-300@SiO_2_ under solvothermal conditions (Figure 5a). The powder XRD patterns did not show any main characteristic reflections for COF-300 until three reaction runs, after which they gradually increased with each run (Figure 5b). The increase in the amount of COF-300 attached to SiO_2_ was also evident from the scanning electron microscopy (SEM) images (Figure 5c–f). The BET surface area of COF-300@SiO_2_ was 431 m^2^·g^−1^, while the surface area of the pristine COF-300 was 1033 m^2^·g^−1^.

**Scheme 1:**
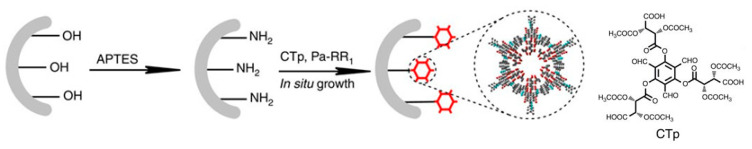
In situ synthesis of chiral COF-bound capillary columns. Reprinted from [41].

An in situ growth approach for the fabrication of chiral COF-containing capillary columns was reported by Yan and co-workers [41]. Tp functionalized with chiral (+)-diacetyl-l-tartaric anhydride, CTp, was pre-polymerized with Pa-1. The fused silica capillary column was modified with APTES, and a solution of pre-polymerized CTpPa-1 was injected into the modified column, which was incubated at 80 °C to in situ synthesize the CTpPa-1-bound capillary column (Scheme 1).

### 2.3. Aluminum Oxide–COF Composites

Aluminum oxide, Al_2_O_3_, is one of the most studied ceramic materials, and it has been used as porous ceramic support in practical applications, such as purification, separation, and catalysis, due to its high mechanical strength, high porosity, low cost, and good chemical and thermal stability [42]. The first report of a COF membrane grown on a commonly used porous α-Al_2_O_3_ ceramic substrate was based on boronic-ester-based COF-5 [42]. Surface functionalization of the porous α-Al_2_O_3_ ceramic support by APTES and subsequent treatment with 4-formylphenylboronic acid rendered a surface modified with boronic acid groups. Then, supported COF-5 membrane was prepared via the co-condensation reaction of 1,4-benzenediboronic acid and 2,3,6,7,10,11-hexahydroxytriphenylene under microwave irradiation. The SEM images indicated that the functionalized α-Al_2_O_3_ support was completely covered with COF-5 with ≈1 µm membrane thickness, without visible defects or cracks. However, the B-O bonds exhibit poor chemical stability, which leads to rapid decomposition upon exposure to moisture, thereby limiting the practical applications of boronic ester COFs [43]. Following a similar procedure, a structurally stable imine-based 3D COF-320 was grown on the α-Al_2_O_3_ support under solvothermal conditions. The COF-320 membrane was homogeneous and compact without defects, with a thickness of ≈4 µm.

**Figure 6:**
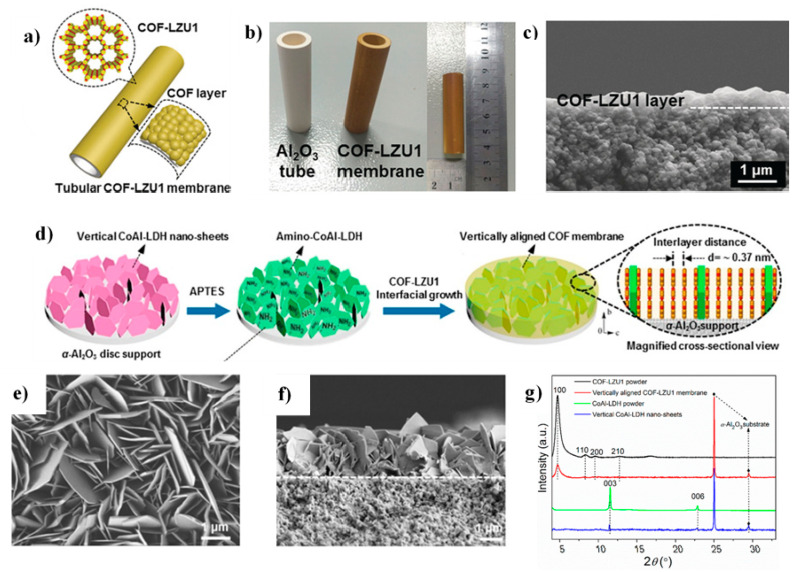
(**a**) Tubular COF-LZU1 membrane; (**b**) photographs of an untreated Al_2_O_3_ tube and a tubular COF-LZU1 membrane; (**c**) cross-sectional SEM image of a tubular COF-LZU1 membrane; (**d**) schematic illustration of the vertically aligned COF membrane; top-view (**e**) and cross-sectional (**f**) SEM images of the CoAl-LDH layer consisting of vertically oriented nanosheets; (**g**) PXRD patterns of the powders and membranes. (**a**–**c**) Reprinted with permission from [44]. Copyright (2018) Wiley-VCH Verlag GmbH and Co. KGaA, Weinheim. (**d**–**g**): Reprinted with permission from [45]. Copyright (2020) American Chemical Society.

Caro, Meng, and co-workers reported the development of continuous and high-quality imine-linked COF-LZU1 membranes supported on alumina tubes [44]. Commercial ceramic tubes were chosen as substrates to allow for facile scale up and cleaning, and good chemical and thermal stability. The COF-LZU1 membrane was fabricated on the surface of the tube by a typical in situ solvothermal method involving APTES-functionalization of the alumina tubes, decoration with TFB, and COF-LZU1 growth (Figure 6a). The dark-yellow tubes (Figure 6b) were analyzed by SEM, revealing a continuous COF layer with well-intergrown grains with sizes of 100–300 nm without any visible cracks or pinholes. Cross-sectional SEM images (Figure 6c) showed that the thickness of the COF-LZU1 layer was about 400 nm. Recently, the 2D COF layers were grown in a vertically aligned manner within a skeleton of vertically aligned CoAl-layered double hydroxide (LDH) layers, which served as a template for COF-LZU1 growth (Figure 6d) [45]. The aminated platelets of the ordered CoAl-LDH layer, perpendicular to the α-Al_2_O_3_ surface, served as a skeleton for the in situ growth of COF-LZU1. In this manner a continuous and defect-free membrane was formed, with ca. 2 µm membrane thickness, having straight vertical channels between the 2D COF layers (Figure 6e,f).

### 2.4. Titanium Dioxide–COF Composites

**Scheme 2:**
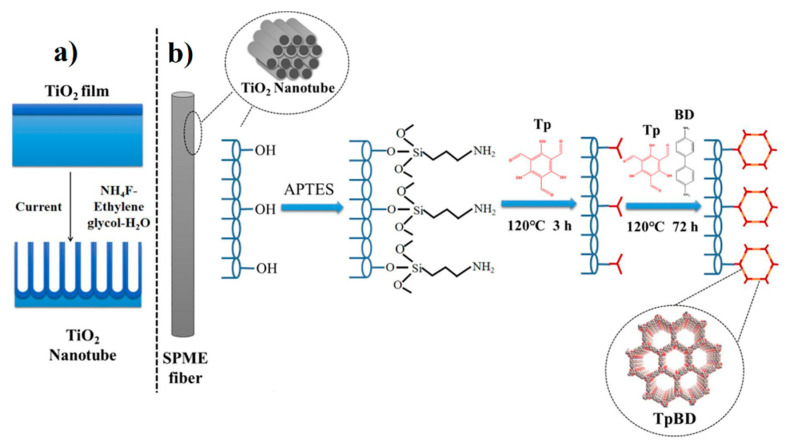
Schematic illustration of (**a**) electrochemical anodization on the surface of titanium wire; (**b**) preparation of TpBD–TiO_2_ solid-phase microextraction fiber. Reprinted with permission from [46]. Copyright (2020) Elsevier Ltd, Amsterdam, The Netherlands.

Titanium is a metal with high corrosion resistance and high stability, and easy surface modification and high chemical activity [46]. The surface of titanium can be modified via anodization to titanium dioxide, TiO_2_, nanotubes, which have provoked widespread interest due to their long-term chemical stability, low cost, facile synthesis, and relative non-toxicity [47]. TpBD COF was covalently bonded on titanium wire via titanium dioxide nanotube arrays and used for solid-phase microextraction of phthalates in vegetables [46]. TiO_2_ nanotubes on the surface of the titanium wire, prepared via electrochemical anodization (Scheme 2a), provided Ti-OH moieties for direct modification with APTES, followed by Tp functionalization, and COF growth under solvothermal conditions (Scheme 2b). Spherical TpBD particles were observed by SEM on the surface of the fiber, the estimated thickness of the coating being 15–20 µm.

### 2.5. Graphene-Based COF Composites

Graphene is a hexagonal honeycomb 2D material formed by sp^2^ hybridization of carbon atoms, forming the thinnest possible atomically flat layers that are stacked via weak van der Waals interlayer forces [48,49]. Due to the thinness of the graphene layer, it can be flexible with bending, twisting, and other deformation modes [49]. The in-plane electrical and thermal conductivities are the highest among known materials, but the through-thickness properties are very poor for stacked graphene. In addition, it exhibits good physicochemical stability and excellent electrical, optical, and mechanical properties, and it has been applied in many fields related to analytical chemistry, such as nanofiltration, separation, and absorption [48].

To gain access to an adsorbent material with both radiation- and acid-resistant properties, a graphene-synergized (GS) TpDAAQ COF composite was fabricated from graphene, Tp, and 2,6-diaminoanthraquinone (DAAQ) via solvothermal method [48]. The obtained GS-COF was then post-synthetically modified to oxime-containing *o*-GS-COF by treatment with hydroxylamine hydrochloride. The SEM images showed that GS-COF exhibited a lamellar structure, evidencing that the composite retained the laminated structure, but attachment and intercalation effects between graphene and the COF led to changes in the surface and local morphology.

**Figure 7:**
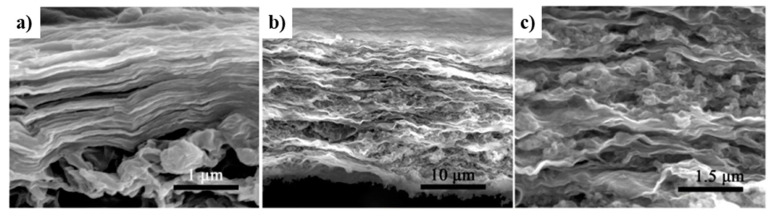
SEM images of GO/COF hybrid membranes: the cross-section of (**a**) a pure GO membrane and (**b**,**c**) a GO/COF 4:1.71 hybrid membrane. Reprinted with permission from [51]. Copyright (2020) Elsevier Ltd.

Graphene oxide (GO) is a graphene derivative, which features a limited amount of oxygen-containing functional groups on its lamellar edges and defective parts [50]. It has attracted increasing interest for desalination, purification, and molecular separation due to its hydrophilicity, nearly frictionless surface, and fast water-transporting channels. Additionally, GO has been widely considered as a candidate for nanofiltration membranes because of its ultrathin 2D structure and excellent tolerance towards harsh chemical environments and organic solvents [51]. The nanochannels between the GO flakes provide pathways for organic solvents and water while blocking larger molecules and ions. However, GO membranes suffer from poor flux of water and organic solvents as well as instability under practical application conditions. To improve the permeate flux, nanostructured GO/COF hybrid membranes with different GO/COF ratios, using COF prepared from TFB and Pa-1 under solvothermal conditions, were prepared by vacuum filtration method. For example, GO/COF 4:1.71 hybrid membrane was fabricated by dispersing 4 mg of GO and 1.71 mg of COF in *N*,*N*-dimethylformamide (DMF), followed by vacuum filtration on a nylon membrane with a pore size of 0.22 µm. The membrane thickness was adjusted by filtrating different amounts of GO and COF while keeping the COF content at 30 wt%, and the flexible hybrid membranes were easily peeled form the nylon substrate. As visualized by SEM, compared with stacked layered structure of GO (Figure 7a), the COF nanoparticles are stacked between the GO layers across the GO/COF membranes (Figure 7b,c).

**Scheme 3:**
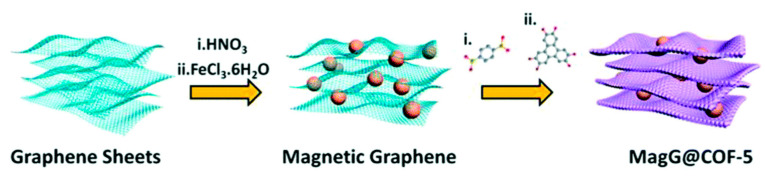
Synthesis of the MagG@COF-5 composite. Reproduced from [53] with permission from The Royal Society of Chemistry.

Hybrid materials based on the combination of graphene with magnetic nanoparticles have drawn intensive interest due to their high surface area, good stability, biocompatibility, unique electrical properties, and strong magnetic response [52,53]. A COF-decorated graphene/magnetite composite, MagG@COF-5, was synthesized by assembling COF-5 layers on the surface of magnetite-decorated graphene (MagG) for recognition of N-linked glycopeptides [53]. The synthetic procedure for the synthesis of MagG@COF-5 is presented in Scheme 3, where MagG is first obtained by graphene functionalization with Fe_3_O_4_ nanoparticles in a hydrothermal reaction, followed by COF-5 growth. The TEM images revealed that after COF coating the graphene nanosheets became thicker and magnetic NPs were fully covered, demonstrating that MagG sheets were successfully coated with COF-5. The BET surface area of MagG@COF-5 was 201 m^2^·g^−1^, higher than that of MagG (65 m^2^·g^−1^). In another study, magnetite-decorated graphene was coated with a polydopamine (PDA) layer to enhance hydrophilicity [54].

Carbon nanotubes (CNTs) are rolled-up graphene sheets, in which the in-plane properties are translated to axial properties, making them among the stiffest axial fibers ever created. CNTs can be either single-walled (SWNTs) or multi-walled nanotubes (MWNTs), which notably affects their mechanical properties [49]. An electroactive COF assembled from 1,3,5-tris(*p*-formylphenyl)benzene (TFPB) and thionine (Thi) was capped with amino-functionalized carbon nanotubes (CNTs) to form COF_Thi_-_TFPB_-CNT composites for sensing [55]. The composite was prepared by simply subjecting the CNTs to the solvothermal COF growth conditions. In electrochemical sensing, the composite structure led to a more uniform and stable electrode surface without losing the catalytic activity of the CNTs.

### 2.6. Metal Nanoparticle-COF Composites

The synthetic approaches to metal nanoparticle-COF composites comprise three main pathways: (i) growth of NPs on pre-synthesized COFs [56,57], allowing for NP growth controlled by the framework, yielding NPs with relatively small sizes, as low as 1.7 nm, as a result of COF pore dimensions. (ii) Growth of COF on pre-synthesized NPs [35,58], where composites containing metal NPs as large as 50 nm have been employed without compromising NP activity and COF crystallinity. These metal NPs have to either be resistant to the COF formation conditions in terms of solvents, acidity, and temperature, or a polymer shell can be first grown under mild conditions, to form a shell around the NPs, protecting them from subsequent COF recrystallization under acidic conditions. (iii) NP precursors are incorporated onto COF building blocks prior to COF synthesis [59], allowing for simultaneous growth of both the COF structure and the NPs. Following this procedure, NPs with size distribution of 12 ± 4 nm have been grown attached to the COF.

First reported strategy for the growth of metal NPs on COFs was carried out by solvent-free gas-phase infiltration of volatile organometallic precursors [60]. In this procedure, volatile precursor [Pd(η^3^-C_3_H_5_)(η^5^-C_5_H_5_)] was diffused into the pores of COF-102, and subsequent treatment by UV-light irradiation gave access to Pd NPs. Average NP size was measured to be 2.4 ± 0.5 nm, comparably larger than the pore diameter of COF-102 of 0.9 nm. However, crystallinity of the network was retained, and therefore, the alignment of the pores was proposed to allow these NPs to grow without compromising the structure.

**Figure 8:**
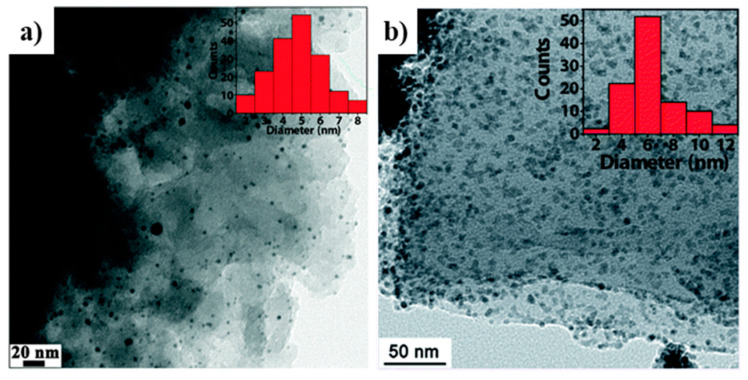
TEM images of (**a**) Au NP-TpPa-1 [56] and (**b**) Pd NP-TpPa-1 [57] composites. Inset image: NP size distribution histogram. (a) Reproduced from [56] with permission from The Royal Society of Chemistry. (**b**) Reproduced from [57] with permission from The Royal Society of Chemistry.

To date, one of the most reported strategies for the synthesis of metal NP-COF composites is simple solution infiltration of NP precursors onto a previously synthesized COF [56,57]. Pioneers were Banerjee and co-workers, who grew gold [56] and palladium [57] NPs on pre-synthesized TpPa-1 by addition of metal NP precursors followed by reduction using NaBH_4_. Metal precursor, HAuCl_4_ or Pd(OAc)_2_, was uniformly dispersed throughout the TpPa-1 framework through interactions mainly with nitrogen and oxygen atoms. Subsequent reduction by NaBH_4_ resulted in a loading of approximately 1.2 wt% Au NPs on TpPa-1, with complete reduction of HAuCl_4_ while preserving the chemical composition of the network [56]. However, a slight loss in crystallinity was observed after treatment with the reducing agent, and it was shown that treatment with NaBH_4_ alone leads to reduction in COF pore size and surface area, highlighting that it is important not to use an excess of the reducing agent [61]. The TEM images showed Au NPs of 5 ± 3 nm uniformly distributed throughout the framework (Figure 8a). Using Pd(OAc)_2_ as precursor, Pd NP loading of 6.4 wt% on TpPa-1 was observed with a size distribution of 7 ± 3 nm (Figure 8b) [57]. However, XRD indicated a slight decrease in the peak attributed to π-π stacking, which, along with the larger NP size, could suggest that Pd NPs were incorporated in the interlayer space and inside the COF pores. The resulting composite materials were applied for the reduction of environmental pollutants and other transformations [56,57]. In another study using a similar approach, ruthenium NPs were loaded on a heteroatom-rich acylhydrazone-linked COF-ASB, prepared from condensation of benzene-1,3,5-tricarbohydrazide and benzene-1,4-dicarboxaldehyde, through RuCl_3_ infiltration and NaBH_4_ reduction [62]. A total amount of 4.1 wt% of Ru was loaded onto the framework, and the NPs in the size range of 2-5 nm were homogeneously distributed in the composite without compromising the crystallinity and chemical composition of the COF.

Pt and Pd NPs with narrow size distributions of 1.7 ± 0.2 nm were synthesized on a thioether-containing COF material also using NaBH_4_ as reducing agent [63]. The formation of NPs with such narrow size distribution was attributed to the presence of the thioether chains inside the pores of the COF, onto which the metal NP precursors could to bind, resulting in greater control of NP growth inside the pores and preventing agglomeration beyond the dimensions of the pores. A total Pt content of 34 wt% and a total Pd content of 26 wt%, respectively, were observed for the composite materials. Furthermore, it was demonstrated that the order of the framework is crucial for the synthesis of such small and uniformly dispersed NPs, whereas using an amorphous thioether-containing polymer formed from the same building blocks as those utilized for the COF resulted in a random size distribution of Pt NPs.

Silver NPs have been grown on pre-synthesized COF-LZU1 in a single step by addition of Ag in the form of AgNO_3_ in deionized water at room temperature [64]. The success of this straightforward strategy was attributed to the high affinity of the Ag ions to nitrogen, as has been observed for other ions, such as copper [65]. Reduced BET surface area indicated that the NPs were successfully dispersed either inside or on the surface of the COF, corresponding to 2.8 wt% of the composite [64].

Strategies consisting of COF growth on pre-synthesized NPs result in the confinement of the metal NPs within a COF shell. In this method, pre-synthesized NPs are subjected to COF growth conditions [35,58]. However, harsh conditions used for COF formation, such as elevated temperature and highly acidic medium, may hinder the use of this methodology for some NP classes.

**Figure 9:**
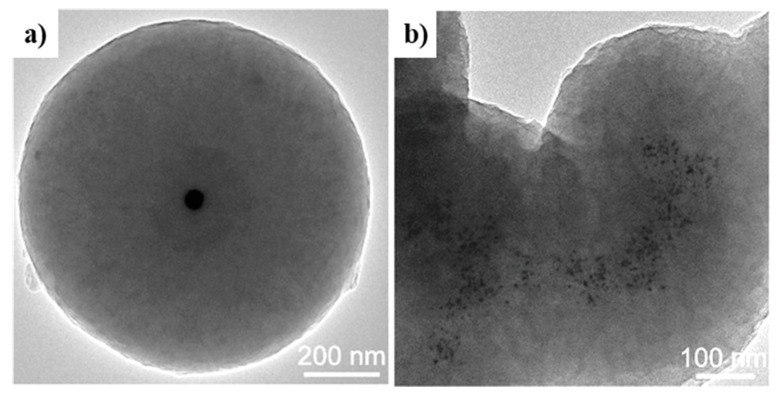
TEM image of (**a**) 0.38 wt% 50 nm Au NP (dark middle circle) and (**b**) 3.8 nm Pt NPs within the COF. Reprinted with permission from [58]. Copyright (2017) American Chemical Society.

Gold and platinum NPs were successfully encapsulated in TAPB-DMTP-COF [58] by pre-functionalization of the NP surface with polyvinylpyrrolidone (PVP), used as a stabilizer in the NP synthesis [58,66], followed by COF growth [58]. Following this strategy, on one hand, a single Au NP of 50 nm was incorporated within a COF shell (Figure 9a), whereas on the other, a cluster of Pt NPs of 3.8 nm was encapsulated by the COF (Figure 9b). The structure and composition of TAPB-DMTP-COF were not compromised. Furthermore, both the COF material and the metal NP-COF composite featured identical surface areas. Therefore, adsorption capacity of the framework was retained. It was proposed that this procedure can be used for the incorporation of other metal NPs resistant to the acidic COF formation conditions. In contrast, using the same amorphous-to-crystalline approach as for Fe_3_O_4_ composites (for the synthesis, see Section 2.1), Maspoch, Zamora, and co-workers grew COF around Au and Pd NPs by a two-step approach of polymer growth under mild conditions and recrystallization of the polymer into COF [35].

Wang, Hu, and co-workers reported a procedure for the in situ growth of Au NPs on the surface of TAPB-DMTP COF [67]. The synthesized Au NPs were successfully immobilized by electrostatic interactions with the unsaturated amino groups present on the TAPB-DMTP surface. The obtained composite retained the COF crystallinity with a high surface area of 1915 m^2^·g^‒1^. However, the surface area of the composite was lower than that obtained for the bulk COF (2385 m^2^·g^‒1^), which was attributed to the growth of Au NPs also inside the COFs pores, evidencing the difficulty of controlling the nanoparticle growth.

**Figure 10:**
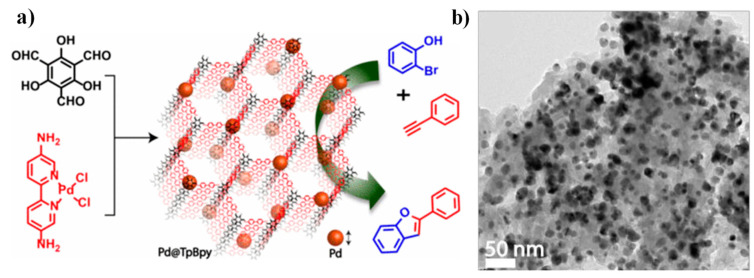
(**a**) Schematic illustration of the composite synthesis and (**b**) a TEM image of Pd NP-TpBpy composite. Reprinted with permission from [59]. Copyright (2017) American Chemical Society.

By pre-functionalizing the building blocks for COF formation with NP precursors, it is possible to simultaneously grow NPs during COF formation, as shown by Balaraman, Banerjee, and co-workers [59]. A bipyridine (Bpy) building block pre-functionalized with PdCl_2_ was employed for the formation of TpBpy (Figure 10), where Pd NPs grew during COF synthesis due to the rupture of the linkage between the NP precursor and the building block. Importantly, the composite was crystalline in nature, showing pore dimensions in the range of 1.4 to 2.3 nm, the latter corresponding to the pore size of TpBpy, which could suggest partial blocking of the pores by the NPs. Total Pd content of 15.2 wt% was found, with Pd NP size being 12 ± 4 nm. Thus, the size of the Pd NPs well exceeded the pore size of the COFs, and it was proposed that the NPs grew into the space between the COF layers and on the surface. Overall, a uniformly dispersed Pd NP-TpBpy composite was formed.

### 2.7. Stainless-Steel COF Composites

For solid-phase microextraction (SPME) composite fibers, stainless-steel wire is usually used as support to load the chosen COF [68]. The most used strategy to fabricate such COF-coated fibers consists of four main steps: (i) pre-cleaning of the stainless-steel wire with aqua regia or hydrofluoric acid to obtain a rough surface; (ii) dip-coating of the raw wire with a thin layer of silicone glue; (iii) careful rotation into COF powder; and (iv) drying/curing. Once cured, the COF-coated fiber can be further coated to protect the COF layer. This strategy provides a chemically and thermally stable coating. In addition to this strategy, in situ COF growth or layer-by-layer synthesis have also been reported. Recently, in situ room-temperature fabrication of TFPB-BD-bonded fiber was reported [69], where an APTES-functionalized stainless-steel wire was treated with one of the COF building blocks, TFPB, and subsequently immersed in a solution containing the other COF building block, BD, and the catalyst acetic acid, leaving the reaction to continue for 3 days at room temperature. In addition, in a layer-by-layer approach [70], the APTES-functionalized stainless-steel wire was immersed alternately in building block solutions containing the organic solvent and the corresponding catalyst. After repeating the process until a desirable thickness (≈5 µm) was reached, the coating was cured by thermal treatment.

### 2.8. Others: COF Composites with Polymeric Substrates and MOFs

**Figure 11:**
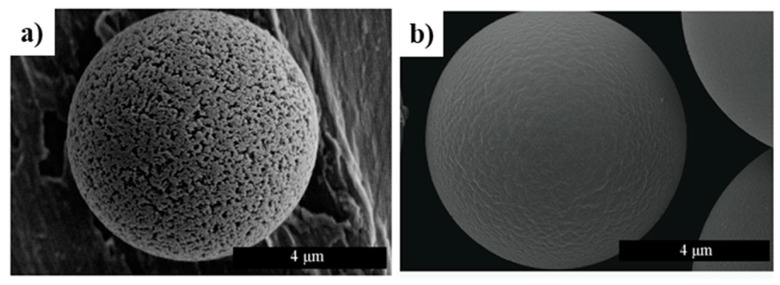
The scanning electron micrographs of (**a**) bare PS-GMA and (**b**) COF@ PS-GMA. Reprinted with permission from [71]. Copyright (2018) Elsevier B.V.

In recent years, the fabrication of polymer-based COF composites has gathered increased attention in solid-phase extraction and separation. To rapidly extract anti-inflammatory drugs from wastewater, a COF-functionalized poly(styrene-divinyl benzene-glycidylmethacrylate) composite, COF@PS-GMA, was prepared by growing COF on the surface of the PS-GMA particles via solvothermal reaction [71]. SEM images (Figure 11) showed that the PS-GMA particles are porous spheres with a size of ≈6 µm, whereas the COF-coated particles exhibit a smooth surface. Additionally, the BET surface area increased from 201 to 404 m^2^·g^−1^ after COF growth.

**Figure 12:**
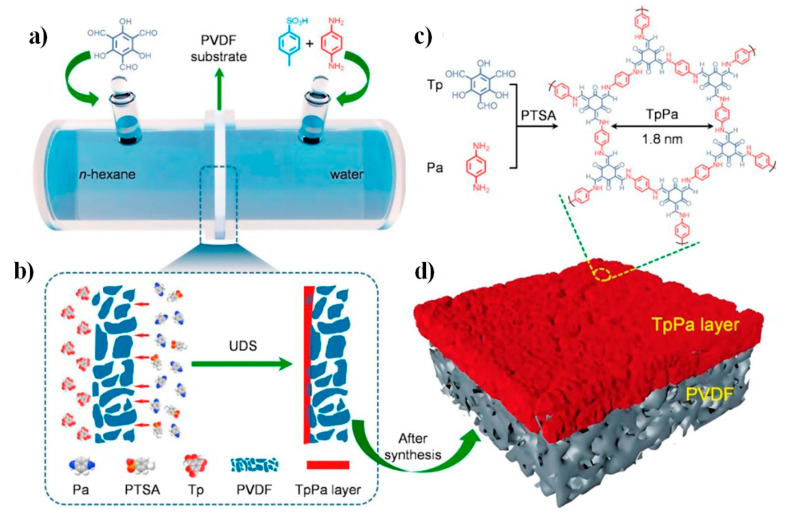
Schematic illustration of the preparation process of the COF-based membranes via the UDS method. (**a**) Schematic illustration of a diffusion cell for the COF growth by UDS. (**b**) Schematic illustration of the formation of the COF layer on the top side of PVDF support via unidirectional diffusion of Pa-1 and PTSA molecules through the pores of the PVDF support to react with the Tp molecules. (**c**) Synthesis of the COF through the condensation of Tp and Pa-1. (**d**) The schematic illustration of intergrowth appearance of COF/PVDF membranes after synthesis. Reprinted with permission from [73]. Copyright (2019) Elsevier B.V.

Polymeric membrane materials are widely used in various separation applications because of their mechanical stability and easy processability [72]. However, physical aging, thermal stability, plasticization, and permeability–selectivity trade-off are limiting the applications of polymeric membranes. To enhance the performance of polymeric membranes, COFs can be used as fillers in the polymer matrix. For the preparation of COF-based composite membranes, unidirectional diffusion synthesis (UDS) of TpPa-1 [73] on commercial polyvinylidene fluoride (PVDF) microfiltration substrates was carried out at room temperature. In the synthesis, an aqueous solution of Pa-1 and a solution of Tp in *n*-hexane were simultaneously charged into the two sides of a diffusion cell (Figure 12). During the reaction, Pa-1 molecules could pass through the macropores of PVDF to react with Tp molecules at the phase interface formed by the solution pair, leading to the in situ synthesis of TpPa-1 crystallites on the top side of the PVDF substrate. A continuous and dense TpPa-1 layer was observed by SEM that was distinctly different compared to the pristine PVDF substrate having a macroporous morphology.

**Figure 13:**
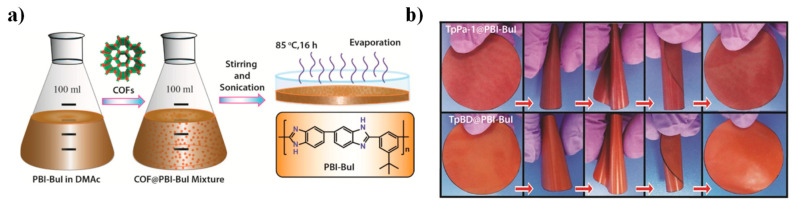
(**a**) Schematic illustration of the preparation of COF@PBI-BuI hybrid membranes. (**b**) Digital photos showing the flexibility of TpPa-1 and TpBD(50)@PBI-BuI hybrid membranes. Reprinted with permission from [72]. Copyright (2016) Wiley-VCH Verlag GmbH and Co. KGaA, Weinheim.

Stable TpPa-1 and TpBD COFs were also used as active phase and incorporated within substituted polybenzimidazole polymer (PBI-BuI) matrix to fabricate self-supported TpPa-1@PBI-BuI and TpBD@PBI-BuI hybrid membranes [72]. Six hybrid membranes were prepared with a sequential increase of COF content in the PBI-BuI polymer (Figure 13). To prepare highly flexible COF(*n*)@PBI-BuI hybrid membranes, where *n* = 20, 40, or 50 wt% of TpPa-1 and TpBD, a solution-casting method using dimethylacetamide (DMAc) as solvent was employed. PBI-BuI solution in DMAc was mixed with the stock suspension of COF. Then, the reaction mixture was poured onto a flat glass surface and heated to 85 °C for 16 h to remove the solvent, after which the membrane was peeled off from the glass surface and dried. In the hybrid membranes, the COF loading could be successfully achieved up to 50%, beyond which defects in the membrane were observed. The average thicknesses of the hybrid membranes were from 47 to 80 µm. The crystallinity of TpPa-1(*n*)@PBI-BuI and TpBD(*n*)@PBI-BuI hybrid membranes was confirmed by wide-angle X-ray diffraction (WAXD). The SEM cross-section of TpBD(*n*)@PBI-BuI hybrid membranes confirmed the distribution of COF particles throughout the membrane matrix. No visible cracks or tears at the COF-polymer interface were seen in the membrane cross-section or the surface. Furthermore, the COF particles were firmly bound within the PBI-BuI backbone, not allowing the COF to leach out from the polymer matrix.

**Figure 14:**
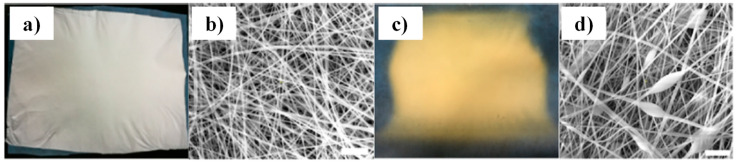
(**a**) Digital photo and (**b**) SEM image of the pure PAN film. (**c**) Digital photo and (**d**) SEM image of the PAN@COF film. Reprinted with permission from [74]. Copyright (2020) American Chemical Society.

Electrospinning has been used to gain access to COF-based films [74]. Dual-pore COF was first synthesized using 4,4′,4″,4‴-(ethane-1,1,2,2-tetrayl)tetraaniline (ETTA) and [1,1′-biphenyl]-4,4′-dicarbaldehyde (BPDA) as monomers, and dispersed homogeneously in DMF with polyacrylonitrile (PAN) to obtain the spinning solution. Then, the PAN@COF nanofiber films with different COF-loading ratios were prepared using a co-electrospinning method. Randomly distributed spindle-shaped COF particles could be clearly observed in the SEM images of the PAN@COF film (Figure 14). XRD characterization indicated that the COF crystals remained intact in the films. Furthermore, the films maintained the dual-pore structure of the COF and showed good stability and excellent reusability in removing phytochromes from vegetable extracts by filtration or immersion.

Mixed matrix membranes (MMMs) using TpPa-2 COF as nanofiller were prepared for water treatment by Xu and co-workers [75]. To prepare the MMMs, TpPa-2 prepared from Tp and 2,5-dimethyl-1,4-phenylenediamine (Pa-2) by microwave (MW) synthesis or mechanochemical (MC) method was blended with polysulfone (PSf) via non-solvent induced phase inversion. The resulting solution was casted onto a glass plate with a thickness of 150 µm, and the casting film was immersed in deionized water for 12 h. The TpPa-2(MW)/PSf membrane had a much smoother surface and less fouling accumulated on the surface compared to the TpPa-2(MC)/PSf membrane. The cross-sectional SEM images showed that nanoparticles with a diameter of about 200 nm were uniformly embedded in both TpPa-2(MW)/PSf and TpPa-2(MC)/PSf membranes, while the pristine PSf membrane was neat, although an asymmetry structure consisting of a dense layer and a macro-void middle region with finger-like pores was observed in all three membranes.

COFs and MOFs are porous materials that have both been used in membrane-based gas separation [76]. However, the fabrication of membranes with both high permeability and high selectivity is a great challenge. To enhance the performance of the membranes, a MOF was grown on a COF membrane to produce COF-MOF membranes. At first, polyaniline (PANI) modified SiO_2_ disks were coated by COF-300 by subjecting them onto COF growth conditions in a Teflon-lined autoclave. Subsequently, the MOF was anchored on top in a similar manner. The SEM images of [COF-300]-[Zn_2_(bdc)_2_(dabco)] composite showed that the thicknesses of the COF and MOF layers were 42 and 55 µm, and in the case of [COF-300]-[ZIF-8], 40 and 60 µm, respectively. In the XRD, only the diffraction peaks of the MOF material were observed, which indicated that the top of the composite membranes was built of pure MOF phase and COF layer was covered completely by it. The COF–MOF membranes showed better selectivity of H_2_/CO_2_ gas mixtures compared to the respective MOF and COF membranes alone.

**Figure 15:**
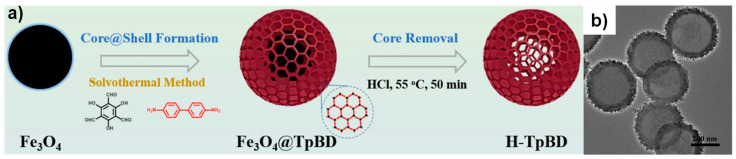
(**a**) Schematic illustration of the synthesis protocols and chemical structures of the H-TpBD. (**b**) TEM image of the synthesized H-TpBD. Reprinted with permission from [77]. Copyright (2018) Elsevier B.V.

Highly water-selective membranes based on hollow COF nanospheres were synthesized using a template-directed method starting from magnetic COF composite Fe_3_O_4_@TpBD (Figure 15a) [77]. The Fe_3_O_4_ core of Fe_3_O_4_@TpBD was etched in HCl solution to yield hollow TpBD (H-TpBD) nanospheres. The H-TpBD nanospheres had a hollow structure with a diameter of around 400 nm and COF shell thickness of around 50 nm (Figure 15b). The H-TpBD nanospheres with well-defined morphology were then incorporated into sodium alginate (SA) polymer to fabricate hybrid membranes for ethanol dehydration. The homogeneous membrane casting solution was spin-cast on PAN substrates to obtain SA-H-TpBD (*X*)/PAN, where *X* (=2, 4, 6, 8, 10) is the mass percentage of H-TpBD to the total of SA and H-TpBD. The field-emission scanning electron microscope (FESEM) images of the membranes showed that the pure SA/PAN membrane had a smooth and dense surface. With the incorporation of H-TpBD, the exposed nanospheres on the membrane surface could be observed. When the H-TpBD content ranged from 2 to 6 wt%, no visible voids at the interface between SA and H-TpBD could be observed. However, when the content was larger than 8 wt%, obvious aggregation of H-TpBD nanospheres was observed. The cross-section images of the membranes showed that for all membranes, the active layers adhered tightly to the porous PAN substrate with a uniform thickness of about 1 µm.

## 3. Analytical Applications

**Figure 16:**
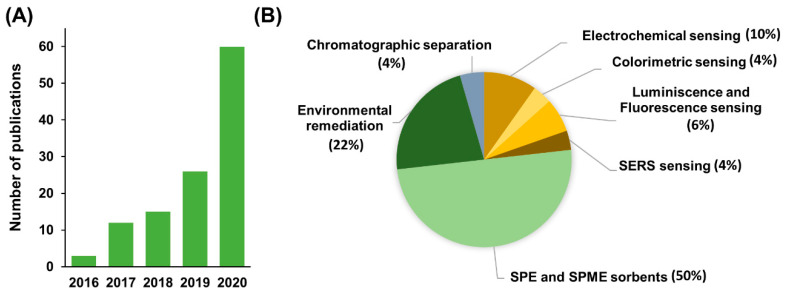
(**A**) Number of publications related to COF composites for analytical applications from 2016-2020 (data obtained from Scifinder^®^ database—August 2020), (**B**) number of publications related to COF composites divided by the analytical application.

The number of reported analytical applications for COF composites has dramatically increased in the last two years (Figure 16A), showing the growing impact that these novel materials have in the field of analytical chemistry. Reported works for COF composites can be classified in two main groups: (i) separation applications, e.g., chromatographic separation, environmental remediation, and extraction techniques, and (ii) chemical sensing, including electrochemical, colorimetric, luminescence, fluorescence, and surface-enhanced Raman scattering (SERS) approaches (Figure 16B). In the following we will highlight selected examples where COF composites have been successfully applied to these two areas.

### 3.1. Applications for Separation

#### 3.1.1. Chromatographic Separation

COF composites have been successfully applied as stationary phases for molecular separations in capillary electrochromatography and HPLC. β-Cyclodextrin combined with TPA-COF loaded on a monolithic organic polymeric column was recently reported for capillary electrochromatographic separation (CEC) of small organic molecules, such as amides, amino acids, nucleosides, aromatic acids, and positional isomers [78]. The hydrophilic β-cyclodextrin/TPA-COF composite provides dipole-dipole and hydrogen-bonding interactions, showing enhanced performance for chromatographic separation in comparison to untreated monolithic polymeric column. Although β-cyclodextrins and COFs as bulk materials usually show high surface area and porosity, when loaded on the monolithic polymeric column, a low surface area of 18 m^2^·g^‒1^ and porosity of 0.034 cm^3^·g^‒1^ were observed, which were ascribed to the polymer chains in the monolith that can fill and block the pores of the β-cyclodextrin/TPA-COF material. The composite showed good reproducibility without significant change in the retention time or resolution after consecutive runs of more than 200 injections. Thus, this work opens a field for further investigation to develop new COF nanocomposites with better physical characteristics for their application in CEC.

COF-based stationary phases have showed excellent performance also in HPLC. To date, different composites based on silica particles coated with 2D COFs, e.g., TpBD [39], chiral-TpPa-1 COF [41], COF-5 [79], and BTA-MTH COF [80], and 3D COFs, such as COF-300 [40], have been reported as hybrid stationary phases. For example, Yan and co-workers [39] reported the preparation of TpBD-coated silica by in situ growth strategy (for composite preparation, see Section 2.2) for the separation of: (i) PAHs as neutral molecules; (ii) hydroquinone, *p*-cresol, and *p*-chlorophenol as acidic molecules; and (iii) nucleobases, nucleosides, and deoxynucleosides as acidic molecules. The separation of the selected molecules was demonstrated to take place inside the pores of the TpBD shell based on size-exclusion effect, and hydrophobic and π-π interactions between the analytes and the COF. Similarly, COF-300 was combined with silica particles to overcome high backpressure problems and enhance the column efficiency in HPLC [40]. COF-300-coated silica was prepared via layer-by-layer method to first form an amorphous polymeric shell, which under solvothermal conditions was transformed into crystalline COF-300 (for composite preparation, see Section 2.2). The silica/COF-300 composite exhibited a higher surface area (431 m^2^·g^‒1^) than the uncoated silica beads (176 m^2^·g^‒1^), showing the effective loading of the COF. The novel stationary phase was successfully applied for the separation of several kinds of analytes, including benzene homologues, PAHs, and nitrophenol, nitroaniline, and aminophenol isomers with high efficiency, selectivity, and precision.

#### 3.1.2. Environmental Remediation

Due to rapid worldwide industrialization together with the increased urbanization there are progressively more issues regarding the alterations of terrestrial and aquatic environments, which directly impact both human and animal health. Anthropological practices associated with industrial activities, daily routines, and even the wrong disposal of commonly used products, such as pharmaceuticals or plastics, contribute to the release of both organic and inorganic contaminants into the environment. Adsorbents based on functional nanomaterials, such as metal oxide nanoparticles [81], carbon-based nanomaterials (carbon nanotubes, graphene, etc.) [82,83], noble metal nanoparticles [84], MOFs [85], and nanocellulose [86], have attracted much attention as “nanotools” for environmental remediation, taking advantage of their high surface area with reactive sites, fast adsorption kinetics, and possibility of tailoring to obtain specific affinity toward targeted analytes. In the recent years, COFs have emerged as advanced nanomaterials for the adsorption and removal of environmental pollutants [22,87]. In comparison to other porous materials, COFs are low-density materials with high crystallinity and large surface areas, designable porosity and pore geometry, and functionality of the building blocks. These advantages, when combined with chemical stability in aqueous medium, render COFs promising materials for water treatment [22]. However, bulk COFs as fine powders are not easy to handle or to separate from the sample matrix due to their low density and small particle size. To this end, to improve their behavior as adsorbents for environmental remediation, COF properties can be tailored with other nanomaterials, e.g., magnetic NPs, graphene, or noble metal NPs, and solid supports, such as cotton fiber (Table 2).

As evident from Table 2, different COF composites have been reported for the separation and recovery of radionuclides, such as uranium [48] and iodine [31,88] from nuclear wastewater and fumes, respectively. For instance, TpDAAQ COF was loaded on graphene sheets to give GS-COF for the adsorption of uranium and plutonium (for more details on the preparation of the composite, see Section 2.5) [48]. GS-COF post-synthetically modified with oxime functionalities afforded a material with high affinity towards uranium with a maximum adsorption capacity of 145 mg·g^‒1^, exceeding the performance of unmodified GS-TpDAAQ (115 mg·g^‒1^) within only 20 min. Compared to other reported adsorbents, GS-COF exhibited better adsorption performance for uranium. For instance, the adsorption capacity of GS-COF (144 mg·g^−1^) was higher than that of GO (92.5 mg·g^−1^) under the same conditions. Furthermore, GS-COF also displayed highly selective adsorption behavior for uranium in the presence of coexisting divalent metal ions and trivalent lanthanide ions.

**Figure 17:**
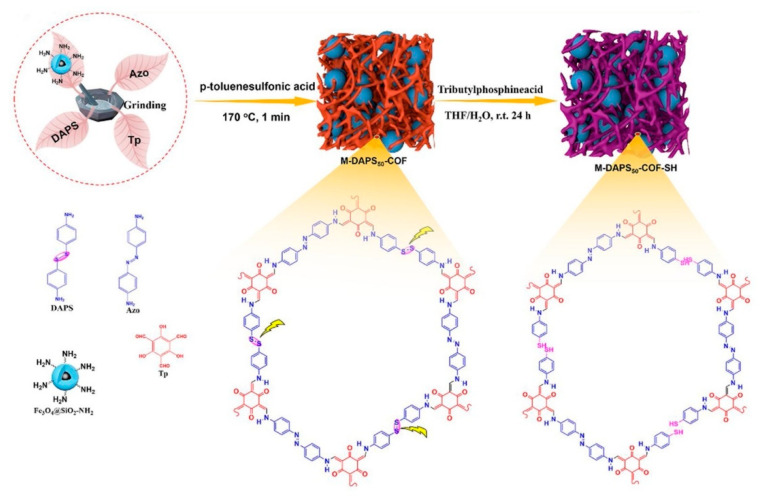
Schematic diagram for the synthesis of Fe_3_O_4_/DAPS-SH composite (reproduced with permission from reference [89], copyright 2020, Elsevier).

The accumulation of heavy metal ions in the aqueous environment has attracted wide concern due to their high toxicity. Recently, different COF composites have been successfully applied for the removal of mercury [64,89], arsenic [90], and chromium [91] from aqueous medium (Table 2). Mercury is one of the most studied heavy metals in the environment due to its high toxicity, high rate of spreading, and bioaccumulation. For the highly efficient removal of mercury from wastewater, Qu and co-workers [64] developed Ag NPs/COF-LZU1 composite (230 m^2^·g^‒1^) that showed high chemical stability even under harsh acidic conditions (pH ≈ 1) (for more details on the composite preparation, see Section 2.6). The COF plays two important roles: on one hand it acts as a support for growing and stabilizing the Ag NPs, and on the other it offers high surface area for adsorbing the target compound and its highly ordered pore channels facilitate the diffusion towards the Ag NPs, where mercury is trapped by amalgamation. The mercury adsorption capacity of Ag NPs/LZU-1 was 113 mg·g^‒1^, outperforming unmodified bulk COF-LZU-1 (24 mg·g^‒1^), demonstrating the high affinity of Ag NPs as mercury scavenger. COFs can be also tailored with thiol or thioether functional groups to provide efficient sites for the capture of mercury due to the strong interaction between sulfide and mercury [97]. Recently, Shuai and co-workers reported a synthetic route for the preparation of a magnetic thiol-functionalized COF composite Fe_3_O_4_/DAPS-SH [89]. The composite was prepared through a one-pot strategy by mixing amine-functionalized Fe_3_O_4_ NPs with the COF components, where in a mixed-linker approach two diamine building blocks, 4,4′-diaminodiphenyldisulfide (DAPS) and *p*-azoaniline (Azo), at different molar ratios were employed (Figure 17). By the incorporation of DAPS, the composite bears thioether (S‒S) functional groups, which were further cleaved into thiol moieties (‒SH). The resulting composite exhibited a maximum adsorption capacity of 383 mg·g^‒1^ for Hg(II) and could be reused at least five times without losses of adsorption efficiency. In addition, adsorption equilibrium was reached rapidly within 10 min, which was attributed to both the high specific surface area of the COF material and the strong affinity between the ‒SH moieties and Hg(II).

Pharmaceuticals and personal care products are a class of compounds considered as emerging contaminants due to their wide use. Their abuse and incorrect disposal may result in the presence of these products and their derivatives in the aquatic environment. Recently, fluorine-functionalized bulk COF was shown to be an efficient adsorbent of ibuprofen from both ultrapure [98] and natural water samples [99]. A step forward in the application of COFs for contaminant removal is the development of magnetic COF composites, which can improve the adsorbent recovery from the sample matrix. Accordingly, different composites have been recently reported as efficient adsorbents to remove diclofenac [32,92,93], sulfonamides [93,94], and triclosan and triclocarban [95] from contaminated water samples (Table 2). For instance, Fe_3_O_4_/TpPa-1 composite was applied as an efficient adsorbent to remove diclofenac sodium from lake water, showing a maximum adsorption capacity of 565 mg·g^‒1^ [32]. However, the surface area of the composite material (352 m^2^·g^‒1^) decreases in comparison to bulk TpPa-1 (704 m^2^·g^‒1^), and therefore the development of improved and easier synthetic routes minimizing the loss of surface area is of high interest. For example, Wang and co-workers reported Fe_3_O_4_/TAPB-DMTA COF composite based on the growth of magnetite NPs directly on the COF obtaining high surface area. This composite was successfully applied for the removal of sulfamethazine with a maximum adsorption capacity of 113 mg·g^‒1^ [93]. Interestingly, the synthesis of the magnetic composite is based on the impregnation of COF pores with iron salts as precursors followed by in situ growth of magnetite NPs. With this approach, no loss in the surface area (2245 m^2^·g^‒1^) was observed when compared to the bulk COF (1958 m^2^·g^‒1^). However, it should be noted that the weak point of this composite is the magnetic behavior, having a saturation magnetization of only 5.2 emu·g^‒1^. For more details of the synthesis of magnetic COF composites, please see Section 2.1.

In another study, Salonen, Espiña and co-workers reported a facile three-step procedure to obtain magnetic TpBD-Me_2_ composite [29]. Under the optimized synthetic conditions, a crystalline composite with 538 m^2^·g^‒1^ was obtained, showing no loss in surface area as compared to bulk TpBD-Me_2_ (468 m^2^·g^‒1^) [100]. Furthermore, taking advantage of the magnetic behavior of the composite, marine biotoxins were adsorbed from seawater with high efficiency, showing calculated maximum adsorption capacities of 812 and 830 mg·g^−1^ for okadaic acid (OA) and dinophysistoxin-1 (DTX-1), respectively. In the case of OA, around a three-fold increase of the adsorption capacity was observed as compared to bulk TpBD-Me_2_ [101], which was attributed to the easier collection of the magnetic adsorbent from the sample matrix that prevents material losses during the procedure. Furthermore, the same magnetic TpBD-Me_2_ composite was very recently applied for the first time as efficient adsorbent to remove endocrine-disrupting pesticides from water [30]. High adsorption efficiencies were found for lipophilic chlorpyrifos and atrazine, with calculated maximum adsorption capacities of 270 and 54 mg·g^‒1^, respectively. On the other hand, polar diquat showed very poor adsorption efficiency of ≈8%, proving the selectivity of the magnetic TpBD-Me_2_ and evidencing that hydrophobic interactions play an important role in the adsorption.

In addition to the above-mentioned contaminants, industrial dyes also pose a serious problem of contamination, making the development of efficient treatment strategies necessary to remove these harmful products from wastewater. In this sense, COF-based membranes have been introduced within advanced water treatment as attractive candidates for nanofiltration of organic dyes [44,73]. For instance, Caro, Meng, and co-workers reported the preparation of a membrane based on the anchoring of COF LZU-1 on alumina support [44] (for details on the composite preparation, see Section 2.3). The membrane showed high water permeance of 760 L·m^‒2^·MPa^‒1^, improving that of commercial nanofiltration membranes, which is usually in the range 10–50 L·m^‒2^·MPa^‒1^. The membrane showed a pore size of ca. 1.8 nm, rendering it remarkably efficient for the separation of high molecular weight dyes, such as methyl blue and Congo red, with rejection rates around 99% showing the efficiency of size exclusion. Additionally, smaller dyes with sizes in the range 1.2–1.6 nm, such as chrome black T, acid fuchsin, and rose Bengal, can also be retained with an efficiency >90%, which was attributed to the presence of staggered pores in the membrane together with the formation of dye micelles resulting in increased sizes. The mechanical resistance of the alumina support in combination with the chemical stability of COF LZU-1 offers membranes with long-term stability that show no decrease in the permeation and retention efficiency even after 80 h of continuous filtration, evidencing their applicability at industrial scale, e.g., in wastewater treatment in textile industries. In another very recent study, Rafiee and co-workers [96] reported a composite based on the combination of MOF-5 with a melamine-based COF, MC5 composite, which showed excellent performance for the efficient removal of auramine-O and rhodamine B (RhB) dyes. Within 8 min, the MC5 composite showed maximum adsorption capacities of 18 and 16 mg·g^‒1^ for auramine-O and RhB, respectively. Although this novel kind of composite still needs further improvement to obtain higher surface active area and to be competitive with other cheaper adsorbents, such as cellulose or active carbon, this study opens a new orientation for developing MOF-COF hybrids for wastewater treatment engineering.

The combination of COFs with metal NPs, such as Au, Pt, Ag, and Pd NPs, provides hybrid catalytic platforms for the degradation of nitroaromatic compounds, such as nitrophenol [56,58,61,63] and organic dyes [61]. For instance, the catalytic behavior of Ag NPs/TPHH COF composite, the synthesis of which is discussed in Section 2.6 [61], was followed by monitoring the UV-Vis spectra of the target molecules. For example, the reduction of 4-nitrophenol (4-NPh) to 4-aminophenol (4-AP) was followed by monitoring the decrease of the absorption peak at 400 nm (4-NPh) and the increase of the absorption peak at 300 nm (4-AP). Only 3 mg of the composite (10.4 wt% Ag) was enough to achieve the complete reduction of 4-NPh at a concentration of around 28 mg·L^−1^ in 100 mL of aqueous solution within 150 s. In addition, Ag NPs/TPHH COF [61] was tested for the reduction of other nitroaromatic compounds, such as 2-nitrophenol, 4-nitroaniline, 1-butoxy-4-nitrobenzene, 4-nitrotoluene, and 4-nitrobenzene, and it showed good catalytic reduction efficiencies, achieving complete reduction of the nitro groups in a few minutes. Additionally, the same composite was tested for the catalytic reduction of dyes, which showed complete degradation within 120-600 s depending on the dye. The Ag NPs/TPHH COF composite demonstrated its effectiveness to catalyze the hydrogenation reaction of -N=N- and -C=N- moieties and degrade the conjugated structure of dyes. Therefore, the development of metal NP-COF catalytic platforms opens new ways for effective environmental wastewater treatment.

#### 3.1.3. Extraction and Microextraction Strategies for Sample Preparation

Sample preparation is the main step in an analytical procedure. In general, the target analytes are at trace or ultratrace level in the sample, making it necessary to separate and preconcentrate them before instrumental analysis. Additionally, separation or extraction from the sample matrix will hinder potential interferents, thereby increasing the sensitivity of the methodology Extraction or microextraction strategies, i.e., solid-phase extraction (SPE) and solid-phase microextraction (SPME), which employ solid adsorbents, are some of the most used strategies for sample preparation because of their simplicity, rapidity, and ability to attain high enrichment factors [102].

**Figure 18:**
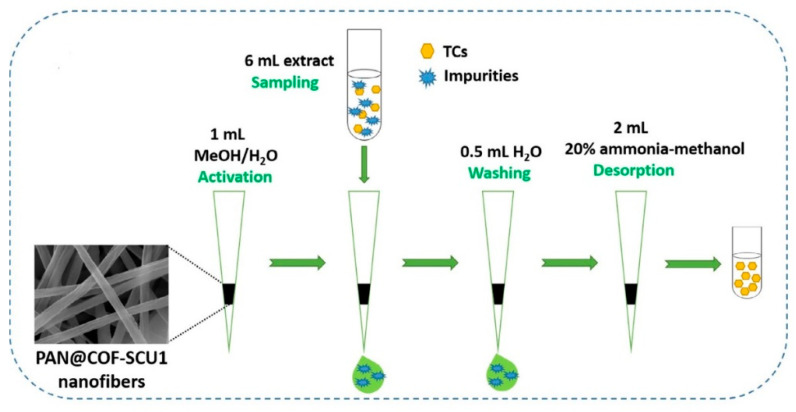
Pipette tip-SPE procedure for the extraction of tetracycline antibiotics (TCs) (adapted with permission from reference [105], copyright 2020, Elsevier).

Regarding COF composites as sorbents in SPE, non-magnetic and magnetic hybrids have been developed depending on the extraction modality employed (Table 3). SPE can be performed in dynamic mode—i.e., the adsorbent is packed into a column and liquid is continuously flowing through the solid sorbent; or in static mode—i.e., the adsorbent is directly dispersed in the sample matrix, keeping the sample volume constant. For example, in the dynamic mode COFs are usually combined with polymers, such as glycidyl methacrylate [71,103] or PAN [104,105]. Such COF composites have been packed into a microcolumn (µSPE), into a syringe (syringe-SPE), or into a pipette tip (PT-SPE). Of these three packing options, PT-SPE is very interesting due to the small amount of adsorbent it requires and thus the sample and solvent consumption is reduced. For example, very recently a novel procedure was reported [105] for the preparation of PAN/TFBPa-1 COF electrospun hybrid nanofibers with TFBPa-1 COF at a loading ratio of 30%. The resulting composite nanofibers were used for the PT-SPE of tetracycline antibiotics in food. To perform the extraction of the analytes, 10 mg of the as-prepared PAN/TFBPa-1 nanofibers were placed into a 0.2 mL pipette-tip using cotton as frits on both sides of the packed composite (Figure 18). The analytes were detected by high-performance liquid chromatography equipped with a photometric diode array detector (HPLC-PAD), and the recoveries of three antibiotics, oxytetracycline, tetracycline, and chlortetracycline, ranged from 82 to 117% with a relative standard deviation of less than 9%. Additionally, the PAN/TFBPa-1 nanofibers can be reused three times without significant loss of extraction efficiency, showing good analytical performance for the analysis of complex matrices.

As mentioned above, SPE can also be performed in static mode, where the adsorbent is directly added to the sample matrix. Both non-magnetic adsorbents (conventional dispersive SPE, dSPE) and magnetic adsorbents (magnetic SPE, mSPE) can be employed. As evident in Table 3, magnetic COFs are by far the most reported composites in this modality. Their main advantage is the easy and rapid collection from the sample matrix by means of an external magnetic field, simplifying the pre-treatment procedure and enhancing extraction efficiency.

In the last few years, different magnetic COFs have been used as suitable adsorbents for mSPE of a wide variety of contaminants, such as paclitaxel [106], PAHs [27,107,108], phthalate esters [34,54,109], bisphenols [110], estrogens [111], phenols [112], aromatic amines [113,114], fluoroquinolones [115,116,117], pesticides [118,119,132,133], plant growth regulators [134], copper [135], and sedatives [120]. The core-shell structure design, where the magnetic core is coated with the COF material, enhances the adsorption performance and avoids the aggregation of the magnetic nanomaterial, making such materials excellent candidates for mSPE. For example, Cai and co-workers [27] developed a facile strategy for the preparation of a magnetic porous nanocomposite combining TpPa-1 with Fe_3_O_4_ NPs (for more details on the preparation of the composite, see Section 2.1). The resulting mTpPa-1 composite was applied for the mSPE of PAHs in tap, lake, and river waters. Under optimal extraction conditions, only 5 mg of mTpPa-1 was enough to extract a mixture of six different PAHs from 200 mL of water sample with recoveries up to 90%, showing the excellent adsorption properties of the composite. After extraction, the PAHs were desorbed in acetonitrile followed by quantitative analysis by HPLC with fluorescence detection, and detection limits in the range of 0.24 to 1.01 ng L^‒1^ were achieved. Furthermore, to add more functionality, polydopamine-coated graphene [54] (for more details on the preparation of the composite, see Section 2.5) and ZIF-8 [120] were hybridized with Fe_3_O_4_/TFBBD, enhancing the hydrophilicity of the composite. The obtained composites were applied for the mSPE of phthalate esters in milk [54] and sedatives in meat samples [120].

The loading of a third component in the magnetic composite can improve the selectivity of the adsorbent towards the target analyte. For example, Li and co-workers [116] reported a Fe_3_O_4_/TpBD composite, which was grafted with Au NPs to facilitate the post-synthetic functionalization with 3-mercaptopropanesulphonate (MPS). Due to the high affinity of Au NPs towards thiols, they provide reactive sites for the immobilization of MPS through the Au‒S bond. The obtained Fe_3_O_4_/TpBD/Au-MPS composite was applied for the selective mSPE of fluoroquinolones in meat samples. Although such samples are indeed very complex matrices, when using the Fe_3_O_4_/TpBD/Au-MPS composite no matrix effect was seen, and recoveries of the target fluoroquinolones in the range of 82–110% were achieved, demonstrating the great purification performance of the functionalized composite to isolate and preconcentrate the analytes.

**Figure 19:**
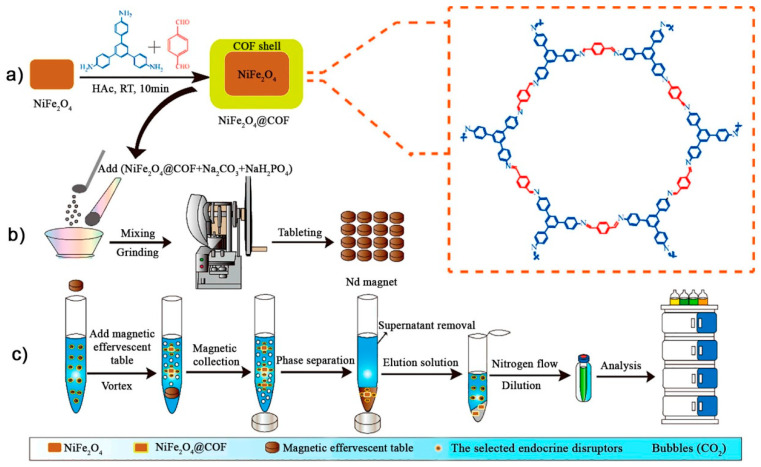
Schematic diagram of the ERME method: (**a**) synthesis of TAPB-TPA-coated NiFe_2_O_4_ composite, (**b**) preparation of the magnetic effervescent tablets, (**c**) pre-concentration and analytical analysis of endocrine disruptors. (Adapted with permission from reference [24], copyright 2020, Elsevier).

Novel dispersive-solvent-free microextraction technique known as effervescence reaction-enhanced microextraction (ERME) was reported using magnetic TAPB-TPA COF composite for the extraction of six endocrine disruptors from environmental and biological samples [24]. The ERME is based on the dispersion of the adsorbent using CO_2_ bubbles generated from an acid/base reaction. The strong volatilization of CO_2_ bubbles from bottom to top in the extraction vial increases the contact areas between the adsorbent and the liquid sample matrix, and thus enhances the extraction recovery (Figure 19). To prepare the effervescent adsorbent, magnetic COF consisting of TAPB-TPA-coated NiFe_2_O_4_ was mixed with sodium dihydrogen phosphate (as acid) and sodium carbonate (as base) in a mortar. Then, the solid mixture was gently pressed to form a tablet. To perform the extraction, the effervescent magnetic composite tablet was placed in contact with the liquid sample. CO_2_ bubbles were immediately generated, which contributed to rapid dispersion of the NiFe_2_O_4_/TAPB-TPA COF composite, decreasing the extraction time to around 3 min and avoiding the utilization of an additional dispersion power, such as vortex or ultrasonication. Furthermore, the composite can be reused to prepare new tablets at least six times without significant loss in extraction efficiency.

The second most widely reported sample treatment strategy using COF composites is the SPME, where the adsorbent is placed onto a stainless-steel wire as a thin layer (Table 3) (for more details on the preparation of the composites, see Section 2.7). SPME can also be performed in two main modes, direct immersion (DI-SPME) or headspace (HS-SPME), depending on the volatility of the target analytes. For sampling using direct extraction, the SPME fiber is placed in the usually liquid sample matrix and stirred for a period of time in order to transport the analytes to the fiber. To date, SNW-1 COF [121], TpPa-NO_2_ COF [122], TpPa-1 [68,123], TFTA-TAPT COF [124], and TpBD [125] were shown to be stable fiber coatings for the DI-SPME of different emerging and persistent organic contaminants in food and environmental samples (Table 3). For instance, Yang and co-workers [124] prepared a stainless steel/TFTA-TAPT COF fiber, which after curing was treated with chitosan and tripolyphosphate to generate a biocompatible protective layer for the COF. Interestingly, this fiber was applied for the DI-SPME of polyfluoroalkyl substances in lake water, and microextraction coupled to analysis by mass spectrometry achieved detection limits in the range of 0.02–0.8 ng L^‒1^.

HS-SPME mode can be used for volatile or semi-volatile analytes. In this approach, the SPME fiber is exposed to the headspace above the sample avoiding direct contact with the sample and thus minimizing the effects associated with interferences present in the sample matrix. To date, different COF-coated fibers have been applied for the HS-SPME of semivolatile analytes, such as pyrethroids [126], polychlorinated biphenyls [69,127], PAHs [70,128], and phthalate esters [46,129] from vegetable, fruit, soil, and water samples. Furthermore, derivatization can be performed to extract non-volatile analytes. For example, Wang and co-workers [130] applied TpBD-coated fiber for HS-SPME of chlorophenols using acetic anhydride as derivatization agent to promote the volatilization of the target analytes and their transport to the headspace.

Since the adsorbent is not in contact with the sample during extraction, complex viscous samples such as honey [121], grind vegetables [46], or soil slurries [127] can be directly placed in the extraction vial without further treatment, simplifying the sample preparation. To favor the transport of the analyte from the sample matrix to the fiber placed in the headspace of the extraction vial, the sample is usually heated at around 50–70 °C under vigorous stirring. Additionally, salinity is an important factor that affects the extraction efficiency. Usually, sodium chloride is added to the sample matrix with the aim of increasing the ionic strength, which worsens the solubility of the analytes in the sample matrix, favoring their transport to the headspace (*salting-out* effect). The fiber is exposed until equilibrium is reached (non-exhaustive extraction). A significant advantage of the SPME extraction is that high enrichment factors (EFs) are achieved, which significantly enhances the sensitivity of the method. For instance, stainless steel (ss)/TFBTPDH composite showed EFs in the range of 307–2327 for pyrethroids in vegetables and fruit [126], ss/TpBD composite provided EFs in the range of 473–1575 for chlorophenols in food [130], ss/TAPB-TMC composite showed EFs in the range of 819–2420 for PAHs in water [128], and TiO_2_/TpBD composite achieved EFs between 226 and 2154 for phthalate esters in vegetables [46]. In addition, for quantitative analysis, gas chromatography coupled to mass spectrometry (GC-MS) is the most used since it is easily coupled to the SPME device (Table 3). The SPME fiber can be injected in the desorption port of the instrument followed by thermal desorption of the extracted analytes.

Recently, Yang and co-workers [69] reported an in situ room-temperature fabrication of a TFPB-BD-bonded stainless-steel fiber achieving good crystallinity with a COF layer thickness of around 10 µm (for more details on the preparation of the composite, see Section 2.7). The novel SPME fiber was applied for the extraction of polychlorinated biphenyls in fish and seafood, reaching detection limits in the range of 0.07–0.35 ng·L^‒1^. In another study, TFPA-TAPP-coated stainless steel fiber was obtained using a layer-by-layer strategy under mild conditions [70], and the as-grown fiber was applied to extract PAHs from water samples by HS-SPME achieving detection limits of 0.006–0.024 µg·L^‒1^.

Another interesting microextraction configuration tested is the so-called thin-film microextraction (TFME), in which the extraction phase, or adsorbent, is configured as a sheet of flat film with high surface-to-volume ratio. With this configuration, the volume of the extraction phase can increase in comparison to conventional fibers. Since the thickness of the extraction phase can be thinner than the fibers, the extraction time is shortened, thereby increasing the extraction rate. Very recently, a novel cellulose/TpBD composite was reported as an efficient adsorbent for the TFME of tetrabromobisphenol-A from water samples [131]. TpBD can be easily embedded in the cellulose matrix, i.e., a mixture of starch and TpBD powder dispersed in water was filtered under vacuum over sheet of filter paper (diameter 7 cm). After filtration, the impregnated paper was pressed overnight. In this study, starch with moderate fluidity and viscosity showed a better performance as a cross-linking agent to immobilize TpBD on the paper compared to neutral silicone glue usually used in the fabrication of composite fibers. To apply the composite as adsorbent, the obtained cellulose/TpBD film was cut into small triangles and directly immersed in the sample solution during 5 min for the extraction of tetrabromobisphenol. Finally, the adsorbed analyte was eluted by continuous dripping of ethanol spray coupled to GC for quantitative analysis. Under optimal extraction conditions, a detection limit of 0.54 µg·L^‒1^ and 50-fold enhancement of the sensitivity were achieved as compared to untreated cellulose paper substrates.

A dual-pore COF based on the combination of ETTA and BPDA building blocks was successfully embedded in PAN matrix by electrospinning (for synthesis, see Section 2.8) [74]. The resulting PAN/COF nanofiber films were applied for TFME of phytochromes and pesticides in food extracts. Briefly, PAN/COF nanofiber film (30 mg, 20 wt% of COF) was directly immersed in the samples for 10 min to reach adsorption equilibrium. After that, the retained analytes were eluted in methanol followed by high-performance liquid chromatography with tandem mass spectrometry (HPLC-MS/MS) analysis. Recoveries of around 99% were achieved for phytochromes, with a maximum adsorption capacity of 0.949 mg·g^‒1^. In the case of pesticides, recoveries in the range 60.5–107.3% were achieved. On one hand, the incorporation of the dual-pore COF provides selective adsorption by size exclusion allowing the removal of the target analytes excluding interferences in complex matrices. On the other hand, PAN matrix provides high chemical stability improving the analyte elution and membrane regeneration by organic solvents. Additionally, recycling studies showed that the PAN/COF nanofiber film can be reused at least ten times without efficiency losses.

These studies open up new avenues for the application of COF-based thin films for the treatment of complex matrices for multiresidue analysis that may be competitive with conventional QuEChERS (quick, easy, cheap, effective, rugged and safe) method in the future.

### 3.2. Applications for Sensing

#### 3.2.1. Electrochemical Sensing

Among the reported applications, electrochemical sensing has gained a lot of attention due to its ease of operation, rapid response, high sensitivity, and low cost. Although metal nanoparticles and carbon-based nanomaterials have been widely used to develop novel surfaces for electrochemical sensors, problems of stability of the electrode surface can lead to poor repeatability. In order to overcome this drawback, current trends of electrochemical sensing are focused on the use of nanomaterials with large specific surface, high porosity, and loading capability, which can be homogenously distributed on the electrode surface. In this sense, COFs have great potential taking advantage of their promising properties. However, the poor inherent electrical conductivity of COFs remains a pitfall in their further application in electrochemistry. Thus, COFs have been successfully combined with highly conductive materials, such as noble metal nanoparticles [67,136,137,138], magnetite nanoparticles [38,139], graphene oxide [140], carbon nanotubes [55,141], or conductive polymers [142], to increase their functionality and broaden their electrochemical applications.

Noble metal nanoparticles (NPs) are the most used in the development of new electrochemical sensors due to their excellent conductivity and catalytic activity. However, for the application of these nanomaterials in the sensing field it is crucial to control their stability, ensuring a good dispersion. In this sense, COFs offer a highly porous support for loading of NPs both inside the pores or attached on the surface. The resulting composites can offer high stability, which enhances the electrochemical performance. Noble metal NPs based on gold, silver, and platinum have been combined with different COF structures for the sensing of chlorogenic acid [67], sodium picrate [136], paraquat [137], and tanshinol [138] in several matrices (Table 4). For instance, Wang, Hu, and co-workers [67] reported TAPB-DMTP@AuNP composite, which was applied as sensor for the determination of chlorogenic acid in coffee, apple, and honeysuckle, displaying a detection limit of 9.5 nM. The sensor exhibited good selectivity towards the target analyte in complex matrices and could be reused for at least 100 cycles without losing its catalytic activity. In comparison with the bare electrode, the composite-modified electrode showed an enhanced electrochemical behavior, which was ascribed to a synergistic effect from the COF active sites and the high conductivity of the Au NPs. In another strategy, Ag NPs were incorporated onto the COF by dispersion of both components in water to obtain the composite TpPa-1@Ag NPs [137]. The Ag NPs bore water-soluble pillar [6] arene (WP6) macrocycles, allowing the immobilization of the Ag NPs on the TpPa-1 surface by H-bonding and π‒π interactions. Furthermore, WP6 enabled selective recognition of the target analyte, e.g., paraquat, showing excellent analytical performance for the determination of this herbicide in lake and tap water with a detection limit of 0.014 µM.

In addition to noble metal NPs, electroactive metal matrices are also useful for the preparation of composites for electrochemical applications. For instance, TpDAAQ was loaded onto a nickel film by drop-casting strategy [143]. Around 1 mg of TpDAAQ was deposited on the nickel substrate (5 × 5 mm) as a very thin layer. The loading of TpDAAQ was observed by an increase in the surface area of the composite (31 m^2^·g^‒1^) in comparison with the bare nickel film (5.5 m^2^·g^‒1^). However, it should be noted that the surface area is much lower as compared to bulk TpDAAQ (≈365 m^2^·g^‒1^) [156]. Although the surface area and crystallinity of the COF in the composite can still be improved, the reported novel sensor was efficiently responsive to hydrazine at low concentrations in lake water and groundwater with a detection limit of 0.07 µM.

Magnetic NPs, such as Fe_3_O_4_, also show strong electrical properties being ideal candidates to be combined with COFs for constructing electrochemical sensors with enhanced properties. In this sense, Fe_3_O_4_ NPs coated with TAPB-DMTP and TpTAPB were reported for the determination of luteolin in food [38] and nitrophenols in water [139], respectively. For obtaining the novel sensors, in both works a dispersion of the corresponding magnetic COF composite was drop-casted on the surface of a pre-cleaned bare glass carbon electrode (GCE). In order to evaluate the behavior of the COF-based sensor, Wang and co-workers [38] tested the electrochemical response to luteolin of bare GCE, GCE coated only with TAPB-DMTP COF, and GCE coated with TAPB-DMTP@Fe_3_O_4_ composite. They observed that just the coating with the COF showed a significant improvement of the signal, which was attributed to the adsorption of the luteolin onto the COF surface by π-π stacking interactions. Furthermore, when bare GCE was coated with the magnetic COF composite, the obtained signal was even more enhanced, demonstrating the synergistic effect between the Fe_3_O_4_ NPs and the COF. On one hand, TAPB-DMTP provided high surface area with active sites to interact with the target analyte, preventing the aggregation of the Fe_3_O_4_ NPs. Meanwhile, Fe_3_O_4_ NPs improved the electron transfer efficiency. Under optimal conditions a low detection limit (7.2 nM) for luteolin sensing in food samples was achieved.

Other highly conductive materials explored for hybridization with COFs to develop new electrochemical sensors are carbon-based nanomaterials, such as graphene oxide [140] and carbon nanotubes [55,141]. Xu and co-workers [141] demonstrated that the combination of TAPB-TPA with CNTs not only improved the conductivity but also offered higher active surface area (147 m^2^·g^‒1^) compared to bulk TAPB-TPA (30 m^2^·g^‒1^). Furthermore, authors expected that the aromatic structure of both composite components together with the NH_2_ functional groups would provide many adsorption sites for interacting with the target analyte, antibacterial agent furazolidone, by π-π stacking interactions, electrostatic interactions, or H-bonding, providing high sensitivity. This was reinforced by an over seven-fold increase in the electrochemical signal using TAPB-TPA@CNT-coated CGE compared to the unmodified electrode. The TAPB-TPA@CNTs composite provided a sensitive platform for furazolidone quantification with a detection limit of 0.0775 µM in chicken and lamb samples.

#### 3.2.2. Luminescent and Colorimetric Sensing

Fluorescence sensing is very attractive due to its high sensitivity, rapid response, and easy operation. In the recent years, two-dimensional (2D) nanomaterials have been put forward for preparing fluorescence and colorimetric composite sensors taking advantage of the high-density of unsaturated atoms on their surface and their high surface area-to-volume ratio [157,158]. In this sense, 2D COFs have been studied to develop hybrid nanomaterials useful for their application as luminescent and colorimetric sensors in the biological and environmental fields (Table 4).

COFs have been considered appropriate tools to fabricate fluorescence sensors due to their conjugated structure. Recently, a core-shell composite resulting from the combination of COF-LZU1 material (shell) with highly fluorescent NPs, such as lanthanide-doped upconversion nanoparticles (UCNPs) (core) was proposed as an ultrasensitive fluorescence sensor for the determination of perfluorooctane sulfonate (PFOS) [144]. The sensing mechanism was based on fluorescence quenching of the UCNPs in the presence of PFOS. The novel composite was applied for the selective detection of PFOS in tap water and food packing with a detection limit of 0.15 pM. The COF-LZU1 shell was identified to play two important roles: (i) selective enrichment of the target analyte taking advantage of the restricted size and chemical environment of the pores, and (ii) stabilization of the lanthanide UCNPs enhancing the fluorescence quantum yield. The authors attributed the enhanced fluorescence intensity of the composite to the formation of ‒C=N‒ bonds in the COF structure, which increases the π-conjugated nature of the core-shell composite, thereby enhancing its fluorescence response. Wang and co-workers [145] described a procedure to fabricate a composite based on carbon dots (CDs) loaded into RhB pre-functionalized PT-DHTA COF. This CDs/RhB@PT-DHTA composite was applied as a mixed fluorescence probe for the detection of trace Hg(II) in water and cosmetic samples with a detection limit of 15.9 nM.

Another interesting manner to develop fluorescence sensors is based on fluorescent COF nanosheets, NUS-30, which were combined with PVDF polymeric matrix to prepare a composite membrane by electrospinning [147]. As expected, NUS-30 nanosheets with few-layer thickness (208 m^2^·g^‒1^) showed lower surface area compared to the bulk NUS-30 powder (1386 m^2^·g^‒1^). Nevertheless, compared to the bulk powder, the NUS-30 nanosheets offered very interesting properties, such as more accessible binding sites (actives sites), amplified fluorescent signal, and negligible aggregation-caused quenching phenomenon, making them appropriate for the development of fluorescence sensors. To enhance the robustness of the nanosheets they were embedded into a polymeric matrix resulting in an easily manageable sensing platform. Fluorescence quenching of PVDF/NUS-30 composite was observed when it was exposed to l-phenylalanine, l-tryptophan, l-alanine, l-threonine, and l-dopa. Among the different tested analytes, l-dopa showed the highest binding affinity, producing a fluorescence quenching around 44% after 10 min of membrane exposure. The PVDF/NUS-30 membrane can be a useful tool for the rapid detection of multiresidue amino acids and small pharmaceutical molecules such as l-dopa. However, the low loading of NUS-30 into the membrane and the lack of selectivity when applied to real biological samples, which are very complex matrices, can result in problems of excluding interfering species from the target analyte/s.

To improve the selectivity of COF composites used as fluorescence sensors, molecularly imprinted polymers (MIPs) have been reported as an attractive option to combine with COFs [148,149]. To develop fluorescence sensors, the hybrid COF/MIP composite can be combined with optosensing nanomaterials such as carbon dots. For instance, Wang and co-workers [148] reported a new fluorescence composite based on CDs coated with a MIP and grafted onto TpPa-1 COF surface (CDs-MIP/TpPa-1) for the selective sensing of 4-ethylguaiacol in wines with a detection limit of 0.11 µM. In the composite, TpPa-1 acts both as an efficient adsorbent that allows the enrichment of the analyte and the exclusion of interfering species taking advantage of the restricted pore size. The enriched analyte is transported to the core of the composite to interact specifically with the molecular cavities of the MIP, producing the quenching of the CD fluorescence. Furthermore, Gao and co-workers [149] reported a fluorescence sensor based on magnetic TFTA-TAPB COF/MIP composite for the sensitive and selective determination of 2,4,6-trinitrophenol (quenching process) in different water samples with a limit of detection of 0.1 nM. To obtain an optosensing platform, the magnetic TFTA-TAPB COF/MIP was grafted with CDs. As in the previously mentioned work, here TFTA-TAPB acts as an efficient adsorbent retaining the analyte. Additionally, the size effect of the COF pores and the specific selectivity of the MIP exclude interferences from the sample matrix. In addition, the presence of a magnetic core facilitates the manipulation and recovery of the composite after each analysis, making it reusable at least for eight times without losing its efficiency.

Colorimetric analysis has been explored as sensing strategy taking advantage of its low cost, simple operation, and good sensitivity, and usually it does not require the use of advanced detection instruments. To date, colorimetric assays using COF-based composites have mainly focused on the combinations with noble metal nanoparticles. Recently, a composite based on the in situ growth of Pd NPs on COF-LZU1 supported onto carboxymethyl cellulose (CMC) was reported for colorimetric detection of HeLa cancer cells in human serum samples [150]. Interestingly, the surface area of the Pd NPs/CMC-LZU1 composite was 403 m^2^·g^‒1^, which is close to the bulk COF-LZU1 surface area (460 m^2^·g^‒1^). To perform the colorimetric assay, the immobilized Pd NPs were functionalized with folic acid (FA), resulting in FA-Pd NPs/CMC-LZU1 composite that is able to bind specifically HeLa cancer cells showing an absorption band at 430 nm. This novel sensor is a promising sensing platform to detect cancer cells from serum samples, with a detection limit of 100 cells mL^−1^.

Furthermore, two different composites based on the in situ growth of Au NPs on COF, i.e., Au NPs/TpBpy COF nanosheets [151] and Au NPs/PTAzo COF [152], were reported as colorimetric sensors for Hg(II) in water samples, with detection limits of 0.33 and 0.75 nm, respectively. In both works, the composite showed stable peroxidase mimetic activity. In the presence of Hg(II), an enhanced peroxidase activity was observed, enabling the oxidation of 3,3′,5,5′-tetramethylbenzidine (TMB) in the presence of hydrogen peroxide with a subsequent color change from colorless to blue. The color intensity derived from the oxidation of TMB is directly proportional to the concentration of Hg in the sample. In addition, due to the high affinity between Hg and Au, both composites showed excellent selectivity with negligible interference from other common ions present in the water samples. In general, the main pitfall of these sensors is the strong interaction between Hg and Au that forms an amalgam, which could limit their reusability due to the harsh conditions, such as thermal treatment, necessary to regenerate the Au NPs [151].

#### 3.2.3. Surface-Enhanced Raman Scattering

SERS has attracted wide attention as an ultrasensitive sensing strategy. Raman scattering can be significantly enhanced using noble metal nanoparticles, Au NPs being the most used in the development of SERS-based sensors. The detection mechanism for these sensing platforms is based on the enhancement of the intensity of Raman scattering from the analytes adsorbed on the surface of the Au NPs or adsorbed in the proximities as a result of the strong local electromagnetic field enhancement [159]. With the aim of stabilizing the metal NP environment to ensure good measurement reproducibility, different materials such as silicon [160], graphene [161], and MOFs [162], have been explored to fabricate hybrid nanostructures. In this sense, COFs are potential SERS substrates to develop noble metal NP/COF composites, since they could exhibit excellent adsorption capability towards both the NPs and the target analyte. To date, Au NPs were combined with different COFs to obtain SERS platforms for the detection of polycyclic aromatic hydrocarbons (PAHs) in water [153], β-lactoglobulin in food [154], and adenosine triphosphate (ATP) in urine [155]. Au NPs/SNW-1 COF composite was applied as a sensitive platform for the recognition of eight different PAHs, such as acenaphthene, phenanthrene, anthracene, fluoranthene, pyrene, and benzo[*a*]anthracene, in tap water and pond water with detection limits in the range 1–0.1 µM [153]. PAHs showed high affinity towards SNW-1 COF through π-π interactions, and the proximity to the immobilized Au NPs produced the characteristic SERS spectrum. The authors demonstrated that the SERS substrate can be applied for sensing mixtures of the target PAHs offering an interesting tool for rapid screening tests. The characteristic peaks of each PAH could be distinguished, and the signal intensity was directly proportional to the concentration. However, multi-residual screening was hampered at low concentrations by the overlap of the peak positions between smaller and larger PAHs.

Li and co-workers [154] reported Au NPs/TAPB-DMTP COF composite as SERS immunosorbent for the detection of β-lactoglobulin protein in food samples with a detection limit of 0.28 nM. The obtained composite showed good crystallinity with a surface area of 900 m^2^·g^‒1^, which is close to the value for the bulk TAPB-DMTP COF (1060 m^2^·g^‒1^). After growing the Au NPs on the COF, the composite was functionalized with protein polyclonal antibody (pAb) as analyte recognition site. The Au NPs/TAPB-DMTP composite mimicked nitroreductase activity with enhanced stability, and was employed as the signal amplification tag to catalyze the reduction of 4-nitrothiophenol (4-NTP) to 4-aminothiophenol (4-ATP) in the presence of sodium borohydride. The produced 4-ATP acts as a bridge to connect the Au NPs generating a Raman “hot-spot.” The COF material not only contributes to the catalytic activity but also offers high adsorption capacity of 4-NTP, allowing high concentration of the substrate around the composite.

## 4. Conclusions and Outlook

In the recent years, COF composites have been broadly used in chromatographic separation, extraction approaches for sample preparation, environmental remediation, and sensing. The dramatic increase in the number of reported analytical applications for these materials evidences their growing impact in the field of analytical chemistry. COF composites offer improved characteristics taking advantage of the synergetic effects between the different composite phases. In addition, as is evident from the wide variety of exciting examples discussed in this review, the area has matured sufficiently to allow for the preparation of COFs in combination with a wide variety of materials through different synthesis strategies. Nonetheless, retaining the high crystallinity and large surface area of bulk COFs in these composites still remains a challenge in many cases, which should be addressed to enable the full utilization of the capacity of these materials. In addition, it should be noted that in many cases synthetic routes are complex and expensive, rendering them difficult to adapt on the industrial scale with the aim of commercialization of COF composites. Further development in the synthesis procedures is necessary to allow for scaling up of the composite materials while retaining their characteristics, to allow these materials to become truly competitive with conventional sorbents.

In terms of extraction applications, COF composites have shown enhanced performances compared to popular carbon-based materials, such as graphene oxide and carbon nanotubes. In addition, in most of the reported works COFs could be regenerated, thereby being reusable several times and reducing costs and waste generation. However, despite the promising results, the practical application of COF composites in real matrices with naturally occurring contaminants at relevant environmental concentrations still needs further improvement and demonstration to fully open up their applications as superior sorbents. Additionally, a better understanding of the adsorption and desorption mechanisms should lead to enhanced design of fit-for-purpose adsorbents that can be reused, avoiding the use of costly and non-ecofriendly organic solvents. This, together with the demonstration of scalability of the materials, would reinforce the usability of COFs in analytical chemistry not only for sample preparation, but also in remediation. On the other hand, COFs have been recently combined with sensors mainly for improved sensitivity. The incorporation of COFs in sensing surfaces as in situ pre-concentration materials could bring great advantages to solving the extremely problematic matrix interferences in biological fluids and environmental applications and making the sensors affordable, if reusability is efficiently demonstrated.

## Figures and Tables

**Table 1:** Abbreviations for commonly used covalent organic framework (COF) building blocks.

Abbreviation	Name
BD	benzidine
BD-Me_2_	*o*-tolidine
BPDA	[1,1′-biphenyl]-4,4′-dicarbaldehyde
Bpy	5,5′-diamino-2,2′-bipyridine
DAAQ	2,6-diaminoanthraquinone
DAB	1,4-diaminobenzene
DHTA	2,5-dihydroxyterephthalaldehyde
DMTP	2,5-dimethoxyterephthalaldehyde
ETTA	4,4′,4″,4‴-(ethane-1,1,2,2-tetrayl)tetraaniline
Pa-1	*p*-phenylenediamine
Tp	1,3,5-triformylphloroglucinol
TAM	tetra(4-anilyl)methane
TAPB	1,3,5-tris(4-aminophenyl)benzene
TAPT	2,4,6-tris(4-aminophenyl)-1,3,5-triazine
TFB, Tb	1,3,5-triformylbenzene
TFPB	1,3,5-tris(*p*-formylphenyl)benzene
TFTA	2,3,5,6-tetrafluoroterephthalaldehyde
TMC	trimesoyl chloride
TPA	terephthaldicarboxaldehyde
TPDA	terephthalaldehyde
TPDH	terephthalic dihydrazide

**Table 2:** Applications of COF composites for environmental remediation.

COF Composite	Surface Area (m^2^·g^‒1^)	Analyte	Maximum Adsorption Capacity (mg·g^‒1^)	Contact Time (min)	Ref
Fe_3_O_4_/TpBD	273	Bisphenol-A	161	5	[23]
		Bisphenol-AF	237		
Fe_3_O_4_/TpBD-Me_2_	538	Okadaic acid	812	120	[29]
		DTX-1	830		
Fe_3_O_4_/TpBD-Me_2_	538	Atrazine	54	120	[30]
		Chlorpyrifos	270		
Fe_3_O_4_/TFBPa-1 COF	872	Iodine	797	n.d.	[31]
Fe_3_O_4_/TpPa-1	352	Diclofenac	565	20	[32]
Al_2_O_3_/LZU-1 COF	n.d.	Organic dyes	n.d.	n.d.	[44]
Oxime-functionalized GS/TpDAAQ COF	51	Uranium	144	20	[48]
Au NPs/TpPa-1	339	Nitrophenol	n.d.	13	[56]
Au NPs/TAPB-DMTP COF	2388	Nitrophenol	n.d.	10	[58]
Ag NPs/TPHH COF	143	Nitroaromatic compounds and organic dyes	n.d.	2‒6	[61]
Pt NPs/ThioCOF	50	Nitrophenol	n.d.	8	[63]
AgNPs/LZU-1 COF	230	Mercury	113	120	[64]
PVDF/TpPa-1	n.d.	Organic dyes	n.d.	n.d.	[73]
Cotton fiber/TAPB-PDA COF	166	Iodine	824	720	[88]
Fe_3_O_4_/DAPS-SH COF	182	Mercury	383	20	[89]
Fe^0^/TAPB-PDA	132	Arsenic	136	720	[90]
Fe_3_O_4_/TpPa-1	485	Chromium	245	60	[91]
Fe_3_O_4_/TAB-DHA	212	Diclofenac	203	30	[92]
Fe_3_O_4_/TAPB-DMTA	2245	Diclofenac	40	50	[93]
		Sulfonamides	55	80	
Fe_3_O_4_/pCOF	31	Sulfonamides	2‒12	20	[94]
Fe_3_O_4_/TPA-TAPB	56	Triclosan	2	20	[95]
		Triclocarban	1.5		
MOF-5/melamine-based COF	7	Auramine-O	18	8	[96]
		Rhodamine-B	16		

COF building blocks: 1,3,5-triformylphloroglucinol (Tp); 2,6-diaminoanthraquinone (DAAQ); 1,3,5-triformylbenzene (TFB); 1,3,5-tris(4-aminophenyl)benzene (TAPB); terephthalaldehyde (PDA); 4,4-diaminodiphenyldisulfide thiol functionalized (DAPS-SH); 1,4-diaminobenzene (Pa-1); 2,5-dihydroxyterephthalaldehyde (DHA); 2,5-dimethoxyterephthalaldehyde (DMTA); terephthaldicarboxaldehyde (TPA); benzidine (BD); *o*-tolidine (BD-Me_2_). GS: graphene sheets; pCOF: porphyrin-based covalent organic framework; DTX-1: dinophysistoxin-1; n.d.: no data.

**Table 3:** Applications of COF composites in extraction and microextraction strategies for sample preparation.

COF Composite	Surface Area (m^2^·g^‒1^)	Analyte	Sample	Extraction Approach	Analytical Technique	LOD	Ref
NiFe_2_O_4_/TAPB-TPA COF	52	Endocrine disruptors	Urine and serum	Effervescent SPE	HPLC-FLD	0.019–0.096 µg·L^‒1^	[24]
Fe_3_O_4_/TpPa-1	248	PAHs	Water	mSPE	HPLC-FLD	0.24–1.01 ng L^‒1^	[27]
Fe_3_O_4_/TpBD COF	n.d.	Phthalate esters	Beverages	mSPE	GC-MS/MS	0.005–2.75 µg·L^‒1^	[34]
TiO_2_/TpBD	899 *	Phthalate esters	Food	HS-SPME	GC-MS/MS	0.001–0.430 µg·L^‒1^	[46]
Fe_3_O_4_/Graphene/COF-5	201	Glycopeptides	Serum	mSPE	MALDI-TOF-MS	0.5 fmol·µL^‒1^	[53]
Fe_3_O_4_/TFBBD COF	176	Phthalate esters	Milk	mSPE	GC-MS	4–20 ng·L^‒1^	[54]
ss/TpPa-1 COF	468 *	Synthetic musks	Water	DI-SPME	GC-MS/MS	0.04–0.31 ng·L^‒1^	[68]
ss/TFPB-BD COF	286 *	Polychlorinated biphenyls	Food	HS-SPME	GC-MS/MS	0.07–0.35 µg·L^‒1^	[69]
ss/TFPA-TAPP COF	n.d.	PAHs	Water	HS-SPME	GC-FID	6.1–24 ng·L^‒1^	[70]
PS-GMA/TFBDABP COF	404	NSAIDs	Water	Syringe SPE	HPLC-UV	0.13–0.82·µg L^‒1^	[71]
PAN/BPDA-ETTA COF	17	Phytochromes	Food	TFME	HPLC-MS/MS	n.d.	[74]
Poly(GMA-DMA)/TMCPa-1 COF	n.d.	Benzophenones	Urine and serum	Microlumn SPE	HPLC-UV	0.4–0.7 µg·L^‒1^	[103]
PAN/SNW-1 COF	205	Sulfonamides	Food	PT-SPE	HPLC-PAD	1.7–2.7 µg·L^‒1^	[104]
PAN/TFBPa-1 COF	47	Tetracycline antibiotics	Food	PT-SPE	HPLC-PAD	0.6–3 µg·L^‒1^	[105]
Fe_3_O_4_/COF-1	n.d.	Paclitaxel	Plasma	mSPE	HPLC-UV	0.02 µg·L^‒1^	[106]
Fe_3_O_4_/LZU-1 COF	n.d.	PAHs	Soil	mSPE	HPLC-UV	0.2–20 ng·L^‒1^	[107]
Fe_3_O_4_/TpBD COF	114	PAHs	Food	mSPE	HPLC-PAD	0.83–11.7 ng·L^‒1^	[108]
Fe_3_O_4_/TFBBD COF	147	Phthalate esters	Plasma	mSPE	GC-MS	2.5–10 ng·L^‒1^	[109]
Fe_3_O_4_/TAPB-TPA COF	181	Bisphenols	Serum	mSPE	HPLC-MS	1–78 ng·L^‒1^	[110]
Fe_3_O_4_/TFBBD COF	202	Estrogens	Urine	mSPE	HPLC-MS	0.2–7.7 ng·L^‒1^	[111]
Fe_3_O_4_/TAPB-TPA COF	179	BPA and BPAF	Tea	mSPE	HPLC-FLD	0.08–0.21 µg·L^‒1^	[112]
Fe_3_O_4_/TpBD COF	171	Heterocyclic aromatic amines	Urine	mSPE	HPLC-MS/MS	0.14–0.46 ng·L^‒1^	[113]
CNTs/CTC-COF	430	Heterocyclic aromatic amines	Food	dSPE	HPLC-MS/MS	5.8–25 ng·kg^‒1^	[114]
Fe_3_O_4_/TFPB-DATP COF	124	Fluoroquinolone antibiotics	Food and serum	mSPE	HPLC-PAD	0.25–0.5 µg·kg^‒1^	[115]
Fe_3_O_4_/TpBD-Au NPs	70	Fluoroquinolone antibiotics	Food	mSPE	HPLC-MS/MS	0.1–1 µg·kg^‒1^	[116]
Fe_3_O_4_/TpPa-1 COF	506	Fluoroquinolone antibiotics	Milk	mSPE	HPLC-UV	0.05–0.20 µg·L^‒1^	[117]
Fe_3_O_4_/SNW-1 COF	157	Benzoylurea pesticides	Wastewaters	mSPE	HPLC-PAD	0.4–1 µg·L^‒1^	[118]
Fe_3_O_4_/TpBD-NO_2_ COF	255	Neonicotinoid pesticides	Food	mSPE	HPLC-UV	0.02–0.05 µg·L^‒1^	[119]
Fe_3_O_4_-ZIF8/TFBBD COF	84	Sedatives	Meat	mSPE	HPLC-MS/MS	0.04–0.2 µg·kg^‒1^	[120]
ss/SNW1 COF	668 *	Phenols	Honey	DI-SPME	GC-MS	0.04–0.5 µg·kg^‒1^	[121]
ss/TpPa-NO_2_ COF	n.d.	Pesticides	Food	DI-SPME	GC-ECD	0.04–0.25 µg·kg^‒1^	[122]
ss/TpPa-1 COF	625 *	Polybrominated diphenyl ethers	Water	DI-SPME	GC-NCI-MS	5.8–22 µg·L^‒1^	[123]
ss/TFTA-TAPT COF	n.d.	Polyfluoroalkyl substances	Water	DI-SPME	nanoESI-MS	0.02–0.8 ng·L^‒1^	[124]
Carbon nitride/TpBD	136	PAHs	Water	DI-SPME	GC/MS	0.02–0.05 µg·L^‒1^	[125]
ss/TFBTPDH COF	722 *	Pyrethroids	Food	HS-SPME	GC-ECD	0.11–0.23 µg·kg^‒1^	[126]
Fe_3_O_4_-TiO_2_/TAPB-TPA COF	167	Polychlorinated biphenyls	Soils	HS-SE	GC-MS	3–6 ng·kg^‒1^	[127]
ss/TAPB-TMC COF	73 *	PAHs	Water	HS-SPME	GC-MS	0.21–0.94 ng·L^‒1^	[128]
ss/rGO-SNW1 COF	116	Phthalate esters	Water	HS-SPME	GC-MS	0.01–0.5 µg·L^‒1^	[129]
ss/TpBD	967 *	Chlorophenols	Food	HS-SPME	GC-MS	0.3–1.8 µg·kg^‒1^	[130]
Cellulose paper/TpBD COF	248 *	Tetrabromobisphenol-A	Water	TFME	PS-MS	0.54 µg·L^‒1^	[131]

COF building blocks: trimesoyl chloride (TMC); 1,4-diaminobenzene (Pa-1); diaminobiphenyl (DABP); 1,3,5-triformylbenzene (TFB); 1,3,5-triformylphloroglucinol (Tp); benzidine (BD); 1,3,5-tris(4-aminophenyl)benzene (TAPB); terephthaldicarboxaldehyde (TPA); *o*-tolidine (BD-Me_2_); 1,3,5-tris(*p*-formyl)benzene (TFPB); 4,4″-diamino-*p*-terphenyl (DATP); 2,5-dihydroxyterephthalaldehyde (Dt); 2,6-diaminoanthraquinone (DAAQ); 2,3,5,6-tetrafluoroterephthalaldehyde (TFTA); 2,4,6-tris(4-aminophenyl)-1,3,5-triazine (TAPT); terephthalic dihydrazide (TPDH); trimesoyl chloride (TMC); tris(4-formylphenyl)amine (TFPA); tetra(4-aminophenyl)porphyrin (TAPP); [1,1′-biphenyl]-4,4′-dicarbaldehyde (BPDA); 4,4′,4′′,4′′′-(ethene-1,1,2,2-tetrayl)tetraaniline (ETTA). GMA: glycidyl methacrylate; EDMA: ethylene dimethacrylate; SPE: solid phase extraction; HPLC-UV: high performance liquid chromatography UV detection; MALDI-TOF-MS: matrix assisted laser desorption ionization-time of flight mass spectrometry; PS-GMA: poly(styrene-divinyl benzene-glycidylmethacrylate): NSAIDs: non-steroidal anti-inflammatory drugs; PAN: polyacrylonitrile; PT-SPE: pipette-tip solid-phase extraction; HPLC-PAD: high performance liquid chromatography with photodiode array detector; mSPE: magnetic-solid phase extraction; PAHs: polycyclic aromatic hydrocarbons; HPLC-FLD: high-performance liquid chromatography fluorescence detection; GC-MS: gas chromatography/mass spectrometry; HPLC-MS: high-performance liquid chromatography-mass spectrometry; HPLC-MS/MS: high-performance liquid chromatography with tandem mass spectrometry; BPA: bisphenol-A; BPAF: bisphenol-AF; CNTs: carbon nanotubes; CTC-COF: cyclotricatechylene based covalent organic framework; dSPE: dispersive-solid phase extraction; ss: stainless steel; DI-SPME: direct immersion-solid phase microextraction; GC-ECD: gas chromatography-electron capture detector; GC-NCI-MS: gas chromatography-negative chemical ion-mass spectrometry; nanoESI-MS: nanoelectrospray ionization-mass spectrometry; HS-SPME: headspace-solid phase microextraction; HS-SE: headspace-sorptive extraction; GC-FID: gas chromatography-flame ionization detector; rGO: reduced graphene oxide; TFME: thin film microextraction; PS-MS: paper spray-mass spectrometry. n.d.: no data * Brunauer-Emmett-Teller (BET) surface area of bulk COF.

**Table 4:** Chemical sensing applications of COF composites.

COF Composite	Synthesis	Sample	Analyte	Sensing Technique	LOD	Ref
TAPB-DMTP COF/Fe_3_O_4_ NPs	Synthesis of COF in the presence of Fe_3_O_4_ nanoparticles	Food	Luteolin	Electrochemical	0.0072 µM	[38]
Thi-TFPB-COF/CNTs	Synthesis of Thi-TFPB-COF in the presence of CNTs.	Drugs	Ascorbic acid	Electrochemical	16.68 µM	[55]
TAPB-DMTP COF/Au NPs	In situ synthesis of Au NPs on TAPB-DMTP COF surface.	Food	Chlorogenic acid	Electrochemical	0.0095 µM	[67]
2HP6@Au-CP6@TpPa-1	Synthesis of TpPa-1 and functionalization with CP6. Assembly of 2HP6@Au NPs on the COF surface.	Water and soil	Sodium picrate	Electrochemical	0.0017 µM	[136]
TpPa-1/WP6@AgNPs	Synthesis of the TpPa-1 and WP6@AgNPs followed assembling of the nanoparticles on the COF surface.	Water	Paraquat	Electrochemical	0.014 µM	[137]
PtNPs@TpBD/MWCNTs	Synthesis of Pt NPs inside the TpBD COF pores, followed by loading onto MWCNTs surface by sonication.	Drugs	Tanshinol	Electrochemical	0.018 µM	[138]
TpTAPB COF/Fe_3_O_4_ NPs	Synthesis of COF in the presence of Fe_3_O_4_ nanoparticles	Water	*p*-nitrophenol	Electrochemical	0.2361 µM	[139]
			*o*-nitrophenol		0.6568 µM	
PN-COF/GO	Synthesis of PN-COF followed by the mixture with GO in DMF	Drugs	Acetaminophen	Electrochemical	0.011 µM	[140]
		Cosmetics	Hydroquinone		0.009 µM	
TAPB-TPA COF/NH_2_-CNTs	Synthesis of COF in the presence of NH_2_-CNTs.	Food	Furazolidone	Electrochemical	0.0775 µM	[141]
COF-1,4/PANi	Loading of monomers ANi and Py inside COF pores followed in situ polymerization.	Air	HCl and NH_3_ gases	Electrochemical	n.d.	[142]
COF-1,4/PPy						
TpDAAQ/Ni	Loading of DQ-COF onto Ni matrix by drop-casting	Water	Hydrazine	Electrochemical	0.07 µM	[143]
UCNPs@LZU1 COF	Synthesis of COF-LZU1 on the surface of amine functionalized UCNPs	Water and food packing	PFOS	Fluorescence	0.15 pM	[144]
CDs/RhB@PT-DHTA COF	First, synthesis of PT-DHTA COF functionalized with rhodamine B. Second grafting of CDs onto the COF surface.	Water and cosmetics	Hg (II)	Fluorescence	0.016 µM	[145]
TFPA-TAPA COF/Au NPs	In situ synthesis of Au NPs on TFPA-TAPA COF nanosheets surface.	n.d.	Hepatitis A virus	Fluorescence	15 pM	[146]
PVDF/NUS-30 COF	Fabrication of PVDF/NUS 30 mixed membrane by electrospinning.	n.d.	Amino acids	Fluorescence	n.d.	[147]
CDs-MIP/TpPa-1 COF	Coating of CNs with a MIP shell and simultaneous loading on COF surface.	Wine	4-ethylguaiacol	Fluorescence	0.11 µM	[148]
Fe_3_O_4_/TFTA-TAPB COF/MIP/CDs	Loading of CDs on the magnetic COF followed in situ polymerization for coating with the MIP (reverse microemulsion method).	Water	2,4,6-trinitrophenol	Fluorescence	0.0001 µM	[149]
FA-Pd NPs/CMC-LZU1 COF	First synthesis of COF-LZU1 in the presence of CMC. Second, in situ synthesis of Pd NPs on the CMC-LZU1 composite. Finally, functionalization of the immobilized Pd NPs with FA.	Serum	HeLa cancer cells	Colorimetric	100 HeLa cells per mL	[150]
Au NPs/TpBpy COF nanosheets	Synthesis of TpBpy COF followed by in situ synthesis Au NPs on the COF surface.	Water	Hg(II)	Colorimetric	0.33 nM	[151]
Au NPs/PTAzo COF	Synthesis of PTAzo COF followed by in situ synthesis Au NPs on the COF surface.	Water	Hg (II)	Colorimetric	0.75 nM	[152]
Au NPs/SNW-1 COF	Self-assembly by mixing the obtained stock solution of SNW-1 and Au NPs under ultrasound.	Water	PAHs	SERS	1–0.1 µM	[153]
Au NPs/TAPB-DMTP COF	TAPB-DMTP COF synthesis followed by in situ growth of Au NPs on the COF.	Food	β-lactoglobulin	SERS	0.28 nM	[154]
Au NPs/BTAPa-1 COF	BTAPA-1 COF synthesis followed by in situ growth of Au NPs on the COF.	Urine	ATP	SERS	0.004 nM	[155]

COF building blocks: 1,3,5-triformylphloroglucinol (Tp); 2,6-diaminoanthraquinone (DAAQ); thionine (Thi);1,3,5-tris(*p*-formyl)benzene (TFPB); benzidine (BD); 1,4-diaminobenzene (Pa-1); 1,3,5-tris(4-aminophenyl)benzene (TAPB); 2,5-dimethoxyterephaldehyde (DMTP); 1,3,5-triformylbenzene (TFB); tris(4-formylphenyl)amine (TFPA); tris(4-aminophenyl)amine (TAPA); 1,3,5-tris(4-aminophenyl) triazine (PT); 2,5-dihydroxyl-terephthalaldehyde (DHTA); 2,3,5,6-tetrafluoroterephthalaldehyde (TFTA); 5,5′-diamino-2,2′-bipyridine (Bpy); benzene-1,3–5-tricarbaldehyde (BTA). GO: graphene oxide; CNTs: carbon nanotubes; PANi: poly(aniline); PPy: poly(pyrrole); UCNPs: upconversion nanoparticles; PVDF: polyvinylidene fluoride; MIP: molecularly imprinted polymer; CDs: carbon dots; FA: folic acid; CMC: carboxymethyl cellulose; ATP: adenosine triphosphate; DMF: dimethylformamide. n.d.: no data.
